# Ferroptosis’s Master Switch GPX4 emerges as universal biomarker for precision immunotherapy: a pan-cancer study with *in vitro* experiments validation

**DOI:** 10.3389/fonc.2025.1643235

**Published:** 2025-10-09

**Authors:** Xiao Li, Min Zhu, Ruihua Dong

**Affiliations:** ^1^ Department of Research Ward, Beijing Friendship Hospital, Capital Medical University, Beijing, China; ^2^ Department of Gastroenterology, Beijing Friendship Hospital, Capital Medical University, State Key Laboratory of Digestive Health, National Clinical Research Center for Digestive Disease, Beijing Key Laboratory of Early Gastrointestinal Cancer Medicine and Medical Devices, Beijing, China

**Keywords:** glutathione peroxidase 4, ferroptosis, pan-cancer, immunotherapy, tumor microenvironment

## Abstract

**Introduction:**

Glutathione peroxidase 4 (GPX4) is a key enzyme in ferroptosis. Gaining insight into GPX4’s mechanisms and biological roles could offer valuable therapeutic insights for cancer treatment.

**Methods:**

By integrating multi-omics data from The Cancer Genome Atlas (TCGA), the Genotype-Tissue Expression Project (GTEx), cBioPortal, the Human Protein Atlas (HPA), UALCAN, Xiaotao platform and et al., we applied systematic bioinformatics approaches to evaluate the expression, prognostic significance, mutation profiles, DNA methylation and tumor immune microenvironment (TIME) infiltration of GPX4 across diverse cancer types. Furthermore, the role of GPX4 in cell proliferation was experimentally validated.

**Results:**

GPX4 was upregulated in several cancer types. Its potential as a diagnostic biomarker was confirmed by its high reliability in differentiating cancerous from normal tissues, with AUC values surpassing 0.8 in multiple cancers. Functional studies verified its oncogenic function in colorectal and gastric cancer cell lines. In terms of prognosis, GPX4 expression levels were closely associated with overall survival across various cancers. Furthermore, we detected a correlation between the mutation burden of GPX4 across different types of cancer and patient survival outcomes. Additionally, immune infiltration analysis showed significant correlations between GPX4 expression and immune cell presence, particularly macrophages and M2 type macrophages. GPX4 expression also correlated highly with immune modulator pathways and checkpoints.

**Conclusion:**

Collectively, these pan-cancer analyses underscore the potential of GPX4 as a therapeutic target and biomarker in multiple cancers. Further indepth studies on GPX4’s regulatory mechanisms and clinicopathological significance are warranted to develop novel therapies for the prevention and treatment of human tumors.

## Introduction

Ferroptosis is characterized as an iron-dependent form of programmed cell death triggered by lipid peroxidation, lethal accumulation of reactive oxygen species (ROS) and subsequent membrane damage. The dysregulation of this process, which is linked to protein degradation pathways, such as autophagy and the ubiquitin–proteasome system, is connected with various pathological conditions, such as tissue ischemia/reperfusion injuries, neurodegeneration and carcinogenesis (1, 2). Increasing evidence suggests that ferroptosis can suppress the progression of various cancers, garnering significant interest due to its potential in tackling hard-to-treat malignancies. In fact, the combination of chemotherapeutic drugs and ferroptosis inducers has been reported in recent years to enhance chemotherapy effectiveness and improve patient outcomes in cancers of the head and neck cancer (3, 4), pancreatic cancer (5) and glioblastoma (6). Therefore, the targeted induction of ferroptosis may represent a novel and promising cancer therapy strategy (7).

The process of lipid peroxidation plays a vital role in ferroptosis. Glutathione peroxidases (GPXs), an enzyme family with peroxidase activity, can protect organisms against oxidative damage by reducing lipid hydroperoxides and hydrogen peroxide (8). GPX4, a member of the GPX family, is the sole isoenzyme capable of reducing phospholipid hydroperoxides (9), and it is pivotal in preventing ferroptosis in cells. Several studies have indicated that GPX4 exhibits a dual role in human cancers, possessing both tumor-suppressive and oncogenic properties (10–12).

Although mounting evidence suggests that GPX4 significantly contributes to the development of certain cancers, its exact role in cancer biology remains to be fully elucidated, and a comprehensive pan-cancer analysis which leverages multi-omics integration, has not yet been performed. Therefore, the aim of this study was to investigate the expression patterns, prognostic significance, methylation status of GPX4, and the potential role of GPX4 in immune therapy across 33 different types of cancer.

## Materials and methods

### The expression profile of GPX4 in pan-cancer

The mRNA expression profiles of GPX4 between the tumor tissues and the corresponding normal tissues across different cancer types were explored using R on Xiantao platform (https://www.xiantao.love/), where the RNAseq data in transcripts per million reads (TPM) format from The Cancer Genome Atlas (TCGA) and Genotype-Tissue Expression database (GTEx) was converted by log2 for analysis and comparison. The Human Protein Atlas (https://www.proteinatlas.org/) was applied to explore the expression levels of GPX4 in tissue through immunohistochemical method. The DNA methylation and protein level of GPX4 in different cancer types was analyzed by CPTAC platform in UALCAN (http://ualcan.path.uab.edu/index.html). We also used the GEPIA2 (http://gepia2.cancer-pku.cn/#index) and the TISIDB website (http://cis.hku.hk/TISIDB/index.php) to examine the connection between the expression level of GPX4 and pathological characteristics of the tumor.

### Survival prognosis analysis

The Cox regression and Kaplan-Meier (KM) survival curves were used to assess the prognostic value of GPX4, including overall survival (OS), disease-specific survival (DSS), and progression-free interval (PFI). Based on data from the TIDE database (http://tide.dfci.harvard.edu), we evaluated the association between methylation levels of GPX4 and OS of patients with different cancers. In TIDE, first, they rank all patients by the molecular level of the gene in the test. Second, at each threshold position, they cut the gene value into 1 (higher than the current threshold) and 0 (otherwise) and run the Cox-PH regression on binary gene values to compute the z-score associated with the target gene covariate. Finally, they select the threshold where the z-score had the same sign (+/-) as the z-score from continuous regression without a cutoff and achieved the largest absolute value (13).

### Evaluation of GPX4 genetic alteration

Using the cBioPortal platform (https://www.cbioportal.org/), genetic alterations of GPX4 across TCGA cancers were identified, encompassing mutations, structural variants, amplifications, and deep deletions. Furthermore, survival analyses were conducted to compare cancers with and without genetic alterations in GPX4.

### Analysis of GPX4 expression profile at single-cell level

At the single-cell level, CancerSEA (http://biocc.hrbmu.edu.cn/CancerSEA/home.jsp) was used to examine the correlation between GPX4 expression and tumor biological functions. To visualize the expression distribution of GPX4 in cancer, the t-distributed stochastic neighbor embedding (t-SNE) diagram was generated.

### Immune evaluation

The association between GPX4 expression and various immune infiltrating cells, such as cancer-associated fibroblasts (CAF), neutrophils, macrophages, B cells, dendritic cells, CD8+ T cells, T-regulatory cells, NK cells, and monocytes, was assessed using TIMER2.0 (http://timer.cistrome.org/). Concurrently, the correlation between GPX4 expression and the expression of immune checkpoint genes was explored using TIDE (http://tide.dfci.harvard.edu).

Additionally, the correlation between GPX4 expression levels and scores related to stroma, immune cells, and the ESTIMATE algorithm was determined using the Sangerbox 3.0 platform (http://www.sangerbox.com/home.html), with the R software package ESTIMATE (version 1.0.13, https://bioinformatics.mdanderson.org/public-software/estimate/) being utilized for the calculations.

### Gene Ontology and Kyoto Encyclopedia of Genes and Genomes analysis

The gene network analysis tool BioGRID (https://thebiogrid.org/) was utilized to examine the networks linked to GPX4. With the top 100 genes that showed significant correlation with GPX4 identified from the GEPIA2 database, GO and KEGG analyses were performed to elucidate the biological and molecular functions of GPX4. The analysis was carried out using the R package.

### Cell culture and transfection

The human colorectal cancer cell line SW480, and the gastric cancer cell line AGS were obtained from the American Type Culture Collection (ATCC; Manassas, VA, USA). SW480 cells were cultured in RPMI 1640 medium (10-040-CV, Corning, Manassas, VA, USA), whereas AGS were cultured in F-12K medium (10-025-CVR, Corning, Manassas, VA, USA), supplemented with 10% fetal bovine serum (A5669701, FBS; Gibco, Paisley, UK), and maintained at 37 °C in a humidified incubator with 5% CO2. Transfections were carried out using Lipofectamine 3000 reagent (L3000-015, Invitrogen, Carlsbad, CA, USA) following the manufacturer’s instructions. After transfection for 48 hours, the cells were used for following assays.

### Western blot

Cells were disrupted using chilled RIPA buffer from Beyotime (P0013, Shanghai, China), and samples were prepared in sodium dodecyl sulfate (SDS) buffer, separated by 12% SDS-PAGE, and then transferred onto a polyvinylidene fluoride (PVDF) membrane. Then, membranes were first blocked with 5% non-fat milk solution for 1 hour at ambient temperature before being incubated with the corresponding primary antibodies (GPX4: 67763-1-Ig, proteintech, China; β-actin: 66009-1-Ig, proteintech, China) at 4 °C for an overnight period. Finally, after 1-hour incubation with HRP-conjugated secondary antibodies (SA00001-1, proteintech, China) at room temperature, protein detection was achieved using Western ECL substrates from BIO-RAD (170-5061, Hercules, CA, USA).

### Cell viability and proliferation assay

For the MTS test, a total of 1000 cells were plated into each well of the 96-well cell culture plates 48 hours post-transfection, with each group having four replicates. To measure cell growth, 20 μL of AQueous Cell Proliferation Assay solution (G3581, Promega, Madison, WI, USA) was added to each well and then incubated at a temperature of 37 °C for a duration of 2 hours. Optical density (OD) readings at 490 nm were taken using a microplate spectrophotometer on day 0 (six hours post-cell seeding) as well as on days 1-3.

The EdU assay was conducted utilizing the Cell-Light™ EdU Apollo^®^643 *In Vitro* Imaging Kit from RiboBio (C10310-1, Guangzhou, China), adhering to the provided guidelines. The treated cells were seeded into 24-well plate 24 hours prior to the assay. EdU was diluted in the culture medium at a ratio of 1:1000 and then incubated for 2 hours. After fixation with a 4% paraformaldehyde solution and permeabilization with 0.5% Triton-100, the cells were incubated with 100μL of 1× Apollo staining reaction solution for 30 minutes in the dark with gentle agitation. After thorough washing, the cell nuclei were stained with Hoechst33342 solution for 30 minutes. Finally, the cellular fluorescence was examined under a fluorescence microscope (Olympus IX51, Japan).

### Statistical analysis

As for *in vitro* experiment result, the graphical results were presented as the mean ± standard deviation (SD) of three independent replicates, and Student’s t-test was performed for the statistical analysis using GraphPad Prism 8.3 software. A p-value < 0.05 was considered to indicate statistical significance. * indicated p < 0.05, ** indicated p < 0.01, and *** indicated p < 0.001.

## Results

### GPX4 was differentially expressed in pan-cancer and shows diagnostic ability

**Figure 1:**
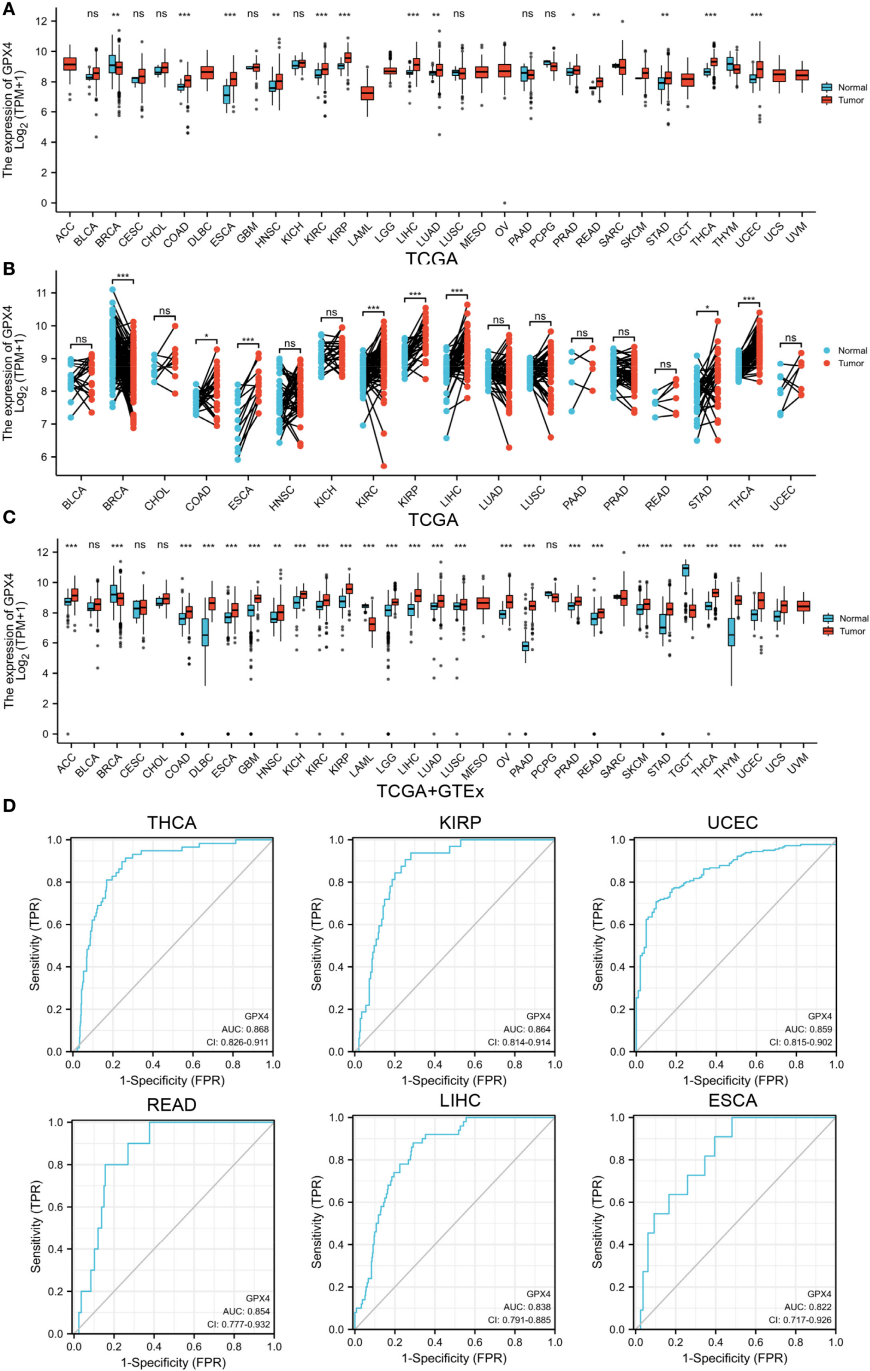
GPX4 expression levels in pan-cancer. **(A)** Expression levels of GPX4 in different human cancers from TCGA datasets. **(B)** Expression levels of GPX4 in paired cancer tissues and adjacent normal tissues from TCGA datasets. **(C)** Expression levels of GPX4 across various human cancers in the TCGA and GTEx databases. ns not significant. *p < 0.05; **p < 0.01; ***p < 0.001. **(D)** ROC of GPX4 in differentiating tumor from normal tissues based on TCGA dataset; FPR, False Positive Rate; TPR, True Positive Rate.

According to TCGA data alone, GPX4 expression was upregulated in colon adenocarcinoma (COAD), esophageal carcinoma (ESCA), head and neck squamous cell carcinoma (HNSC), kidney renal clear cell carcinoma (KIRC), kidney renal papillary cell carcinoma (KIRP), liver hepatocellular carcinoma (LIHC), lung adenocarcinoma (LUAD), prostate adenocarcinoma (PRAD), rectum adenocarcinoma (READ), sarcoma (SARC), stomach adenocarcinoma (STAD), thyroid carcinoma (THCA), and uterine corpus endometrial carcinoma (UCEC), but downregulated in breast invasive carcinoma (BRCA) (**Figure 1A**). Next, we estimated GPX4 expression in paired cancer tissues and adjacent normal tissues using TCGA datasets. GPX4 expression was significantly higher in COAD, ESCA, KIRC, KIRP, LIHC, STAD, and THCA compared to paired adjacent normal tissues, but significantly lower in BRCA (**Figure 1B**). Since normal tissue is limited in TCGA dataset for some types of cancer, we combined data from TCGA and GTEx. In 27 out of 33 cancer types, GPX4 expression differed significantly. The overexpression of GPX4 was observed in several cancers, including adrenocortical carcinoma (ACC), COAD, diffuse large B-cell lymphoma (DLBC), ESCA, glioblastoma multiforme (GBM), HNSC, kidney chromophobe (KICH), KIRC, KIRP, brain lower grade glioma (LGG), LIHC, LUAD, lung squamous cell carcinoma (LUSC), ovarian serous cystadenocarcinoma (OV), pancreatic adenocarcinoma (PAAD), PRAD, READ, SARC, skin cutaneous melanoma (SKCM), STAD, THCA, thymoma (THYM), UCEC, and uterine carcinosarcoma (UCS). However, GPX4 was downregulated in BRCA, LAML, and testicular germ cell tumors (TGCT) compared to normal tissues (**Figure 1C**). Subsequently, we further analyzed protein levels of GPX4 using a large-scale proteome dataset from the National Cancer Institute’s CPTAC dataset. A significant increase in GPX4 expression in lung cancer and a decrease in breast cancer was observed, which was consistent with the transcriptional data (**Supplementary Figure S1A**). Consistent with the sequencing results, the results of immunohistochemistry also support that the expression level of GPX4 in colon cancer and liver cancer was significantly higher than that in the corresponding normal tissues (**Supplementary Figures S1B, C**).

In view of the pathological and clinical feature of GPX4 across different cancer types, we investigated whether GPX4 could be used as a potential biomarker for the diagnosis of human cancers. As shown in **Figure 1D** and **Supplementary Table S1**, GPX4 expression levels were highly reliable in separating cancer from normal tissue, especially in THCA, KIRP, UCEC, READ, LIHC, ESCA, and COAD, where the area under the curve (AUC) exceeded 0.8. Considering that there were a limited number of normal tissues in the TCGA database, we further included the GTEx data into the analysis and found that the discriminating value of GPX4 in some tissues was even improved. AUCs exceeded 0.9 in PAAD, LAML, THYM, TGCT, DLBC, THCA, and LIHC (**Supplementary Table S2**).

We also observed significant correlations between GPX4 expression and disease stage in ESCA, HNSC, THCA, and TGCT (**Supplementary Figure S2A**, **Supplementary Table S3**). Furthermore, analysis using the TISIDB database revealed that expression of GPX4 was associated with tumor grade in HNSC, KIRC, LGG, LIHC, STAD, and UCEC (**Supplementary Figure S2B**).

### GPX4 promoted cancer cell proliferation

**Figure 2:**
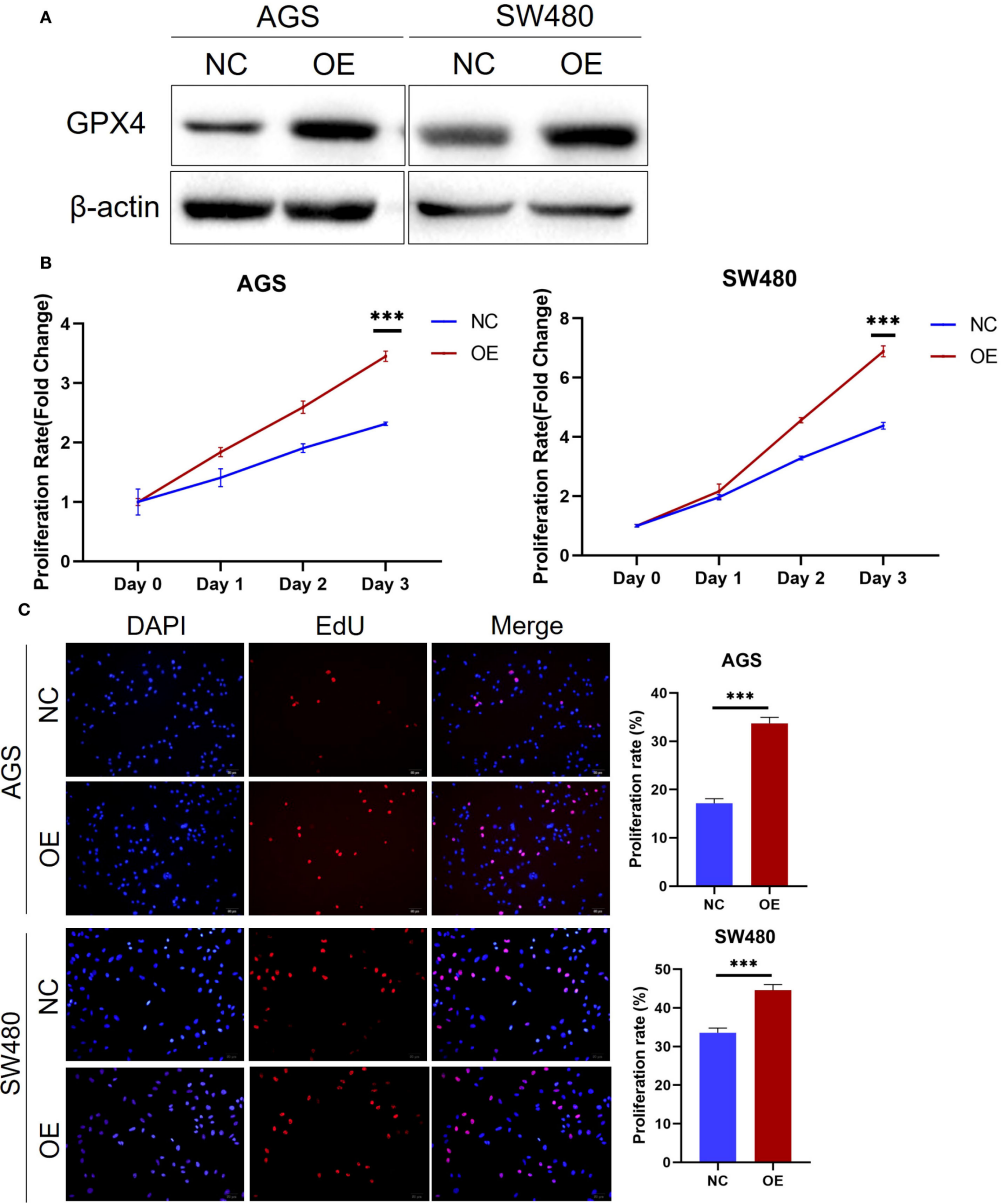
GPX4 promote cancer cell proliferation. **(A)** GPX4 expression level was confirmed by western blot in AGS and SW480 cells after transfection. **(B)** Cell proliferation of AGS and SW480 cells with GPX4 overexpression were determined using MTS assay. ***P < 0.001. **(C)** EdU assay showed overexpression of GPX4 increased the proportion of cells entering the S phase in AGS and SW480 cells. Scale bars, 50μm; ***P < 0.001.

Since GPX4 is highly expressed in various cancer tissues, we hypothesize that it plays an oncogenic role in these tumors. To test this hypothesis, we conducted functional experiments in colorectal and gastric cancer cell lines. Initially, we transfected AGS gastric cancer cells and SW480 colorectal cancer cells and with GPX4 overexpression plasmid, and Western blot (WB) experiments confirmed the efficiency of overexpression (**Figure 2A**). Further functional experiments revealed that GPX4 overexpression significantly promoted the proliferation of AGS and SW480 cells (**Figure 2B**). Concurrently, we observed that GPX4 overexpression increased the proportion of cells in the proliferative phase (**Figure 2C**). The above results suggest that GPX4 plays a pro-oncogenic role in colorectal and gastric cancers.

### The prognostic values of GPX4 in human pan-cancer

**Figure 3:**
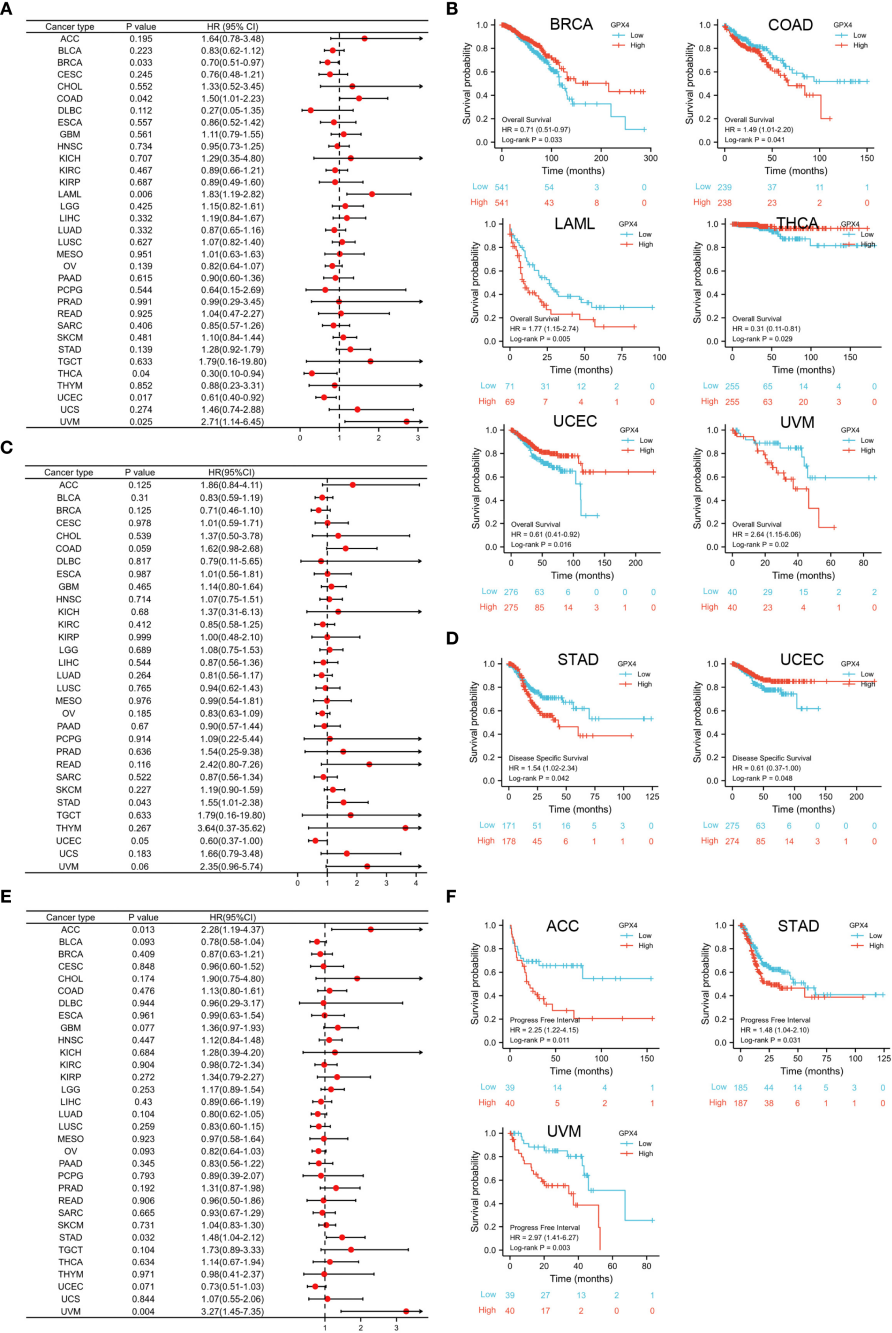
Prognostic value of GPX4 in pan-cancer. **(A)** Cox regression model-based correlation analysis of GPX4 expression with overall survival across various types of cancer.; **(B)** Kaplan-Meier method-based OS curves comparing high and low GPX4 expression levels in various cancer types.; **(C)** Forest plot showed the prognostic role of GPX4 in DSS analysis; **(D)** Kaplan-Meier method-based DSS curves showed the prognostic role of GPX4; **(E)** Forest plot showed the prognostic role of GPX4 in PFI analysis; **(F)** KM method showed the prognostic role of GPX4 in PFI analysis.

The GPX4 gene may serve as an accurate predictor of survival outcomes across a wide range of cancers. According to Cox regression, GPX4 expression levels were significantly associated with OS in six cancer types: BRCA, COAD, LAML, THCA, UCEC, and UVM (**Figure 3A**). The results from the KM survival curves also demonstrated that elevated GPX4 levels were correlated with poorer OS in COAD, LAML, and UVM, but with better OS in BRCA, THCA, and UCEC (**Figure 3B**). In terms of DSS analysis, the forest plot derived from Cox regression outcomes indicated that GPX4 expression had a positive correlation with the hazard ratios for DSS in STAD, but a negative correlation in UCEC (**Figure 3C**). Similarly, KM analysis revealed that increased GPX4 expression predicted worse DSS in STAD patients, but better DSS in UCEC patients (**Figure 3D**). Both Cox regression and KM analyses indicated that high GPX4 expression was linked to poor PFI in ACC, STAD, and UVM (**Figures 3E, F**).

### Genetic alteration analysis of GPX4 in pan-cancer

**Figure 4:**
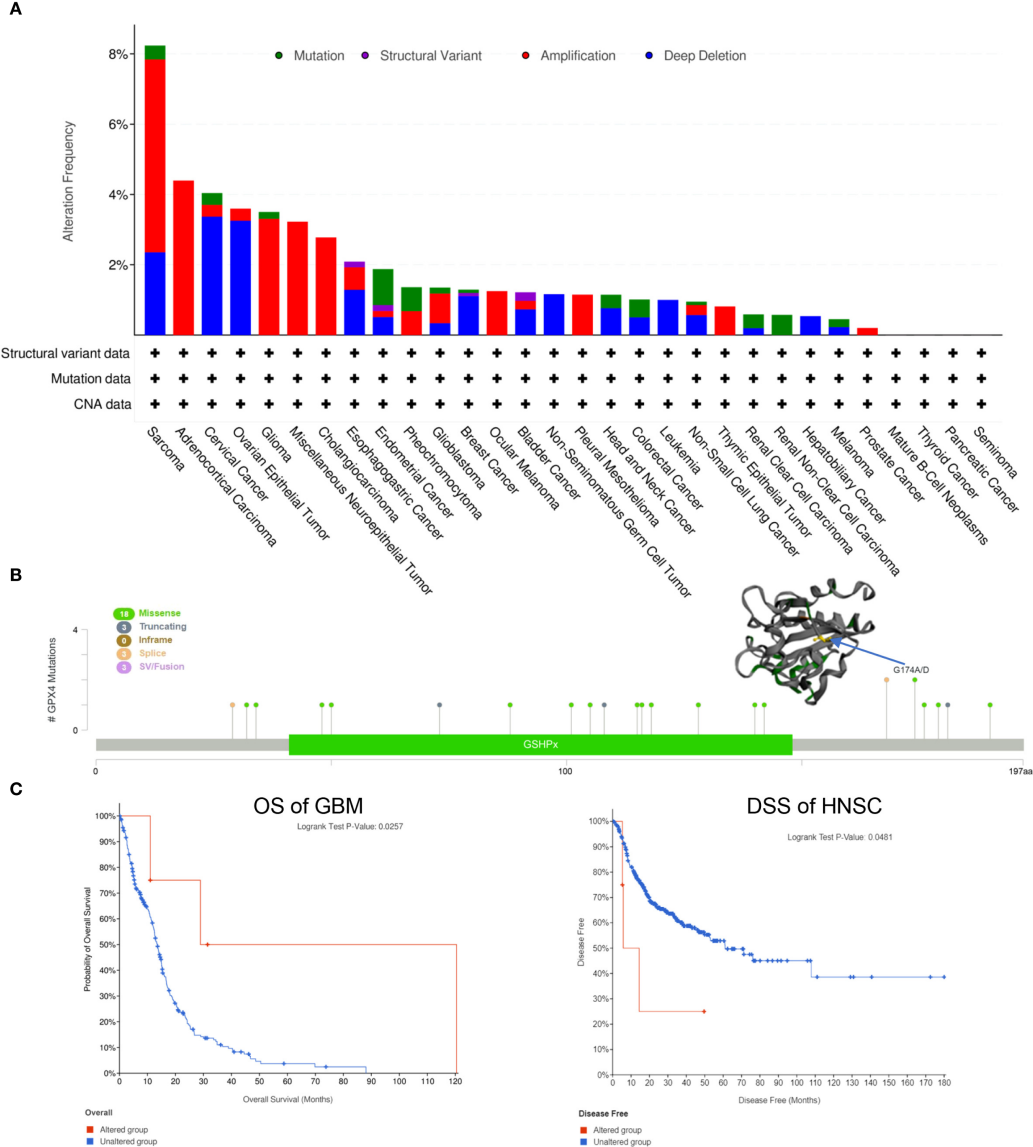
Genetic alteration of GPX4 in pan-cancer. **(A)** The mutational status of GPX4 across various types of cancer was analyzed using the cBioPortal tool.; **(B)** Main mutation types of GPX4 and G174A/D mutation site was visualized in the 3D structure of GPX4 protein; **(C)** Roles of GPX4 alteration in the patients’ prognosis in GBM and HNSC.

Using the cBioPortal web server, we investigated genetic alterations of GPX4 across a range of cancers. As depicted in **Figure 4A**, SARC had the highest rate of GPX4 mutations, approximately 8%, with amplification being the predominant form of genetic alterations. ACC, Glioma, miscellaneous neuroepithelial tumors, and cholangio carcinoma (CHOL) also showed amplification-dominated variants. In OV and cervical squamous cell carcinoma and endocervical (CESC), both female reproductive system cancers, deep deletion emerged as the most prevalent type of mutation.

Subsequently, we investigated the mutation types and mutation sites within the GPX4 genomic sequence. A total of 27 mutations were identified, with missense mutations being the most common (18 cases). Truncating mutation, splice mutation, and SV/fusion mutation were each observed in 3 cases. Notably, the truncating mutation G174A/D could potentially be a driver mutation in cancers (**Figure 4B**). A genetic alteration in GPX4 was associated with improved overall survival in patients with GBM (p=0.0257) (**Figure 4C**). In contrast, HNSC patients with GPX4 genetic alteration displayed a poor DSS (p=0.0481), although no significant was observe in OS (p=0.3870; **Figure 4C**). In other cancer types, GPX4 genetic alterations did not significant effect OS or DFS (**Supplementary Figure S3**).

### The methylation levels of GPX4 in pan-cancer

**Figure 5:**
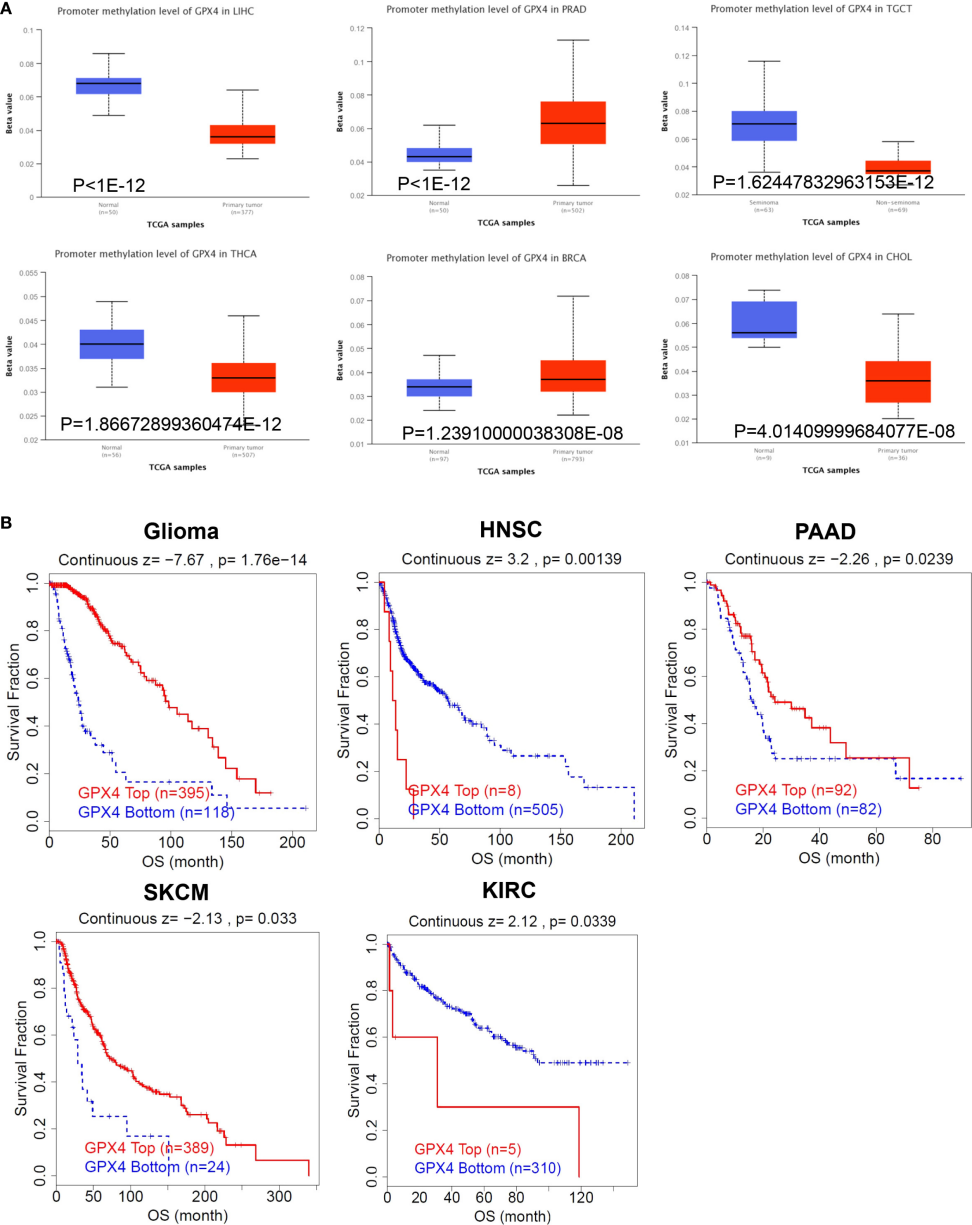
The methylation levels of GPX4 in pan-cancer. **(A)** The UALCAN database displayed the methylation levels of GPX4 in cancers, and TOP 6 cancers with the most significant P-values were shown; **(B)** The correlation between GPX4 methylation levels and overall survival across various types of cancer.

It has been established that alterations in DNA methylation patterns influence gene expression (14). Consequently, we investigated the methylation levels of GPX4 across TCGA pan-cancers utilizing the UALCAN database. Compared to normal samples, GPX4 promoter methylation levels were found to be decreased in LIHC, TGCT, THCA, CHOL, READ, LUAD, KIRP, BLCA, ESCA, GBM, and PAAD. In contrast, PRAD, BRCA, and PCPG exhibited significantly elevated GPX4 promoter methylation levels compared to their normal counterparts (**Figure 5A**, **Supplementary Figure S4A**). Moreover, no significant alterations in GPX4 methylation levels were observed in other types of cancer (**Supplementary Figure S4B**). These findings imply that promoter methylation of GPX4 might be accountable for the dysregulated expression of GPX4 in these cancers.

Subsequently, we utilized the TIDE database to assess the relationship between methylation levels and survival outcomes. As shown in **Figure 5B**, an increased methylation level of GPX4 was associated with improved OS in glioma, PAAD, and SKCM, but with poorer OS in HNSC and KIRC.

### The expression pattern of GPX4 at the single-cell level

The CancerSEA database was used to investigate the expression pattern of GPX4 at the single-cell level and its functional implications in cancer biology. As summarized in **Supplementary Figure S5A**, GPX4 expression was correlated with various functional states across cancers. Specifically, in NSCLC, GPX4 expression showed negative correlations with DNA damage, DNA repair, and the cell cycle, but positive correlations with metastasis, angiogenesis, inflammation, epithelial–mesenchymal transition (EMT), and quiescence (**Supplementary Figure S5B**). In UM, GPX4 expression was negatively associated with quiescence, DNA damage, apoptosis, DNA repair, metastasis, hypoxia, invasion, and differentiation (**Supplementary Figure S5C**). The single-cell expression distributions of GPX4 in NSCLC and UM are further displayed in **Supplementary Figures S5D** and **S5E**, respectively. Based on these findings, we propose that GPX4 plays a critical role in cancer development and progression.

### The roles of GPX4 in the immune infiltration in pan-cancer

**Figure 6:**
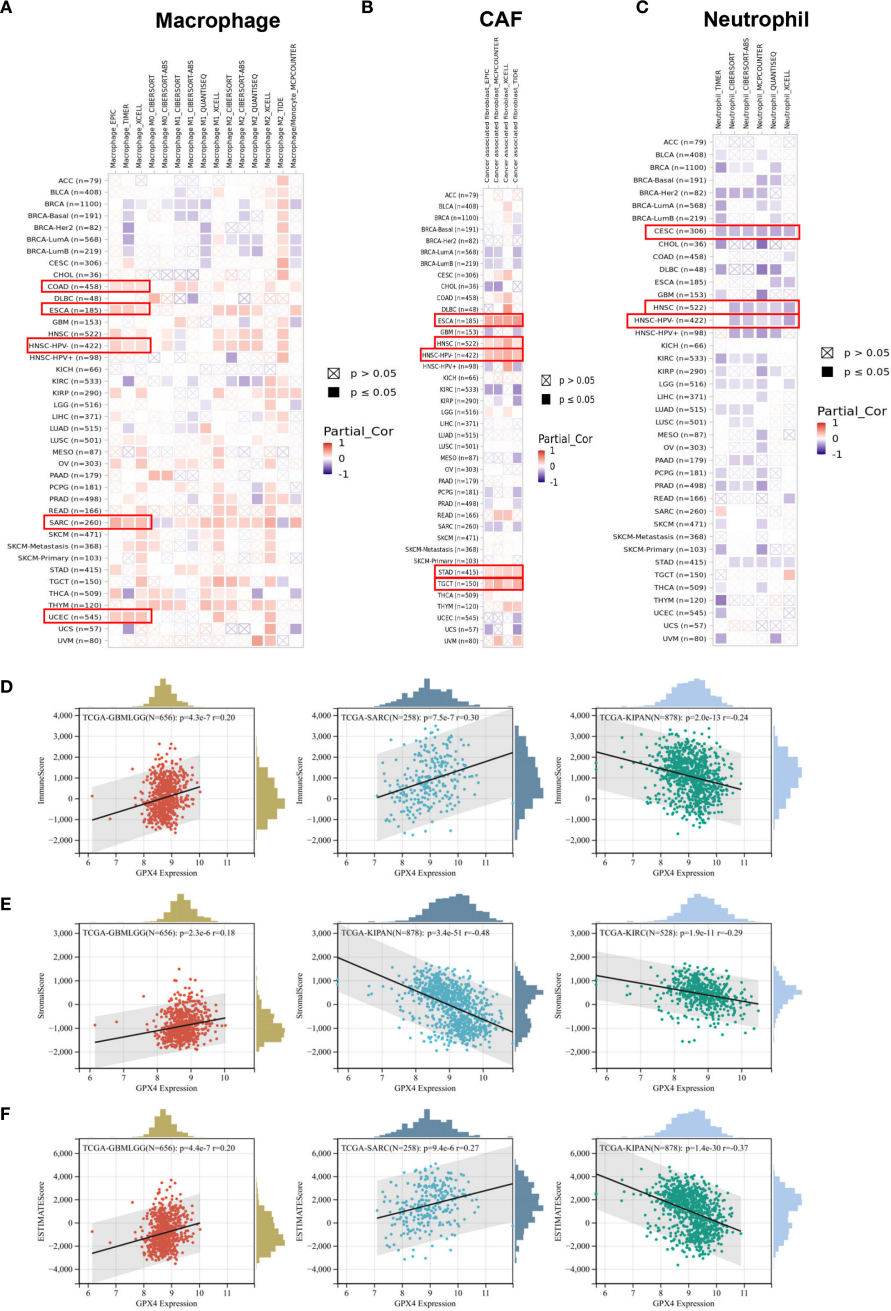
The correlation between the GPX4 expression levels and tumor infiltrating immune cells. **(A–C)** The TIMER2.0 database depicted the relationship between GPX4 expression and immune infiltration of B cell **(A)**, cancer-associated fibroblast **(B)**, and T cell CD8+**(C)** through several algorithms. **(D–F)** The top three cancer types with the most significant correlation between the GPX4 expression and Immune score **(D)**, Stroma score **(E)**, and ESTIMATE score **(F)**.

Through comprehensive analysis, we investigated the potential relationship between GPX4 expression and tumor-infiltrating immune cells. Using seven computational algorithms including TIMER, EPIC, MCPCOUNTER, CIBERSORT, CIBERSORT-ABS, QUANTISEQ, and XCELL, we evaluated immune cell infiltration levels across all TCGA tumor types. A strong association was observed between GPX4 expression and macrophage infiltration in COAD, DLBL, ESCA, HNSC-HPV^-^, SARC, and UCEC. Notably, ESCA, HNSC-HPV^-^, and SARC exhibited infiltration of M2 type macrophages, suggesting a positive link between GPX4 expression and the presence of M2 macrophages (**Figure 6A**). Additionally, GPX4 expression was significantly correlated with the infiltration of cancer-associated fibroblasts (CAFs) in ESCA, HNSC, STAD, and TGCT, neutrophils in CESCs and HNSCs, B cells in HNSC-HPV^+^ and TGCT, CD8+ T cells in HNSC-HPV+, and regulatory T cells (Treg) in HNSC (**Figures 6B, C**, **Supplementary Figures S6A–C**). However, no significant correlation was observed between GPX4 expression and the infiltration of dendritic cells (DCs), natural killer (NK) cells, CD4+ T cells, monocytes, or other immune cell types (**Supplementary Figures S6D–I**).

Furthermore, we also computed immune and stromal scores using the ESTIMATE algorithm (**Supplementary Table S4**). GPX4 expression showed the most significant correlation with immune scores in GBMLGG, SARC, and pan-kidney cancer (KIPAN) (**Figure 6D**). Regarding stromal scores, significant correlations between GPX4 expression and stromal scores were observed in GBMLGG, KIPAN, and KIRC (**Figure 6E**). As shown in **Figure 6F**, GPX4 expression was positively correlated with ESTIMATE scores in GBMLGG and SARC, but negatively correlated in KIPAN.

### Correlations between GPX4 expression and immune modulator pathways and genes associated with immune checkpoints

We next investigated the correlation between GPX4 expression and immune modulator pathways as well as genes associated with immune checkpoints using data from the TCGA database. The heatmap displayed that GPX4 expression was highly correlated with the levels of numerous chemokines and chemokine receptors, such as CXCL16, CCL28, CX3CL1, CCL5, CCR10, CXCR5, and CXCR3, across the majority of cancer types (**Supplementary Figure S7**). Furthermore, the expression of GPX4 was closely associated with MHC molecules, immune activation genes, and immunosuppressive genes such as HLA-A, HLA-B, CD160, TNFRSF18, TNFRSF4 and CD276 in most cancer types. Subsequent analysis also demonstrated a clear relationship between GPX4 expression and key immune checkpoints such as VEGFB, TGFB1, CD276, TNFRSF18, and TNFRSF4 across most cancer types (**Supplementary Figure S7**).

### The relationship between GPX4 expression and Tumor Mutational Burden and Microsatellite Instability

TMB and MSI are comprehensive genetic markers employed to forecast the effectiveness of immune checkpoint inhibitors (ICIs). Thus, we assessed the correlation between GPX4 expression and both MSI and TMB. GPX4 expression levels were positively correlated with TMB in UCEC, LUAD, and STES, but negatively correlated with TMB in LAML and HNSC (**Supplementary Figure S9A**). In UCEC, PRAD, THCA, LIHC, STES, and KIPAN, a positive correlation was observed between GPX4 expression and MSI, whereas a negative correlation was found in GBMLGG (**Supplementary Figure S9B**).

### Potential therapeutic values of GPX4 in immunotherapy cohorts

The therapeutic potential of GPX4 in cancer was investigated using publicly available datasets. Across 21 immunotherapy cohorts, GPX4 exhibited predictive capability for response to immune checkpoint-targeted therapy in 10 cohorts, with AUC values exceeding 0.5. Notably, in the Gide 2019 cohort, the predictive AUC for GPX4 was 0.83, surpassing the MSI score, TIDE, CD274, CD8, T. Clonality, and B. Clonality (**Supplementary Figure S10**). Moreover, GPX4 has been demonstrated prognostic value in patients undergoing immune checkpoint blockade therapy. Higher GPX4 expression was associated with improved clinical outcomes in melanoma trials involving immune checkpoint inhibitors (**Supplementary Figure S11**).

### The network of protein-protein interactions and enrichment analysis of GPX4

**Figure 7:**
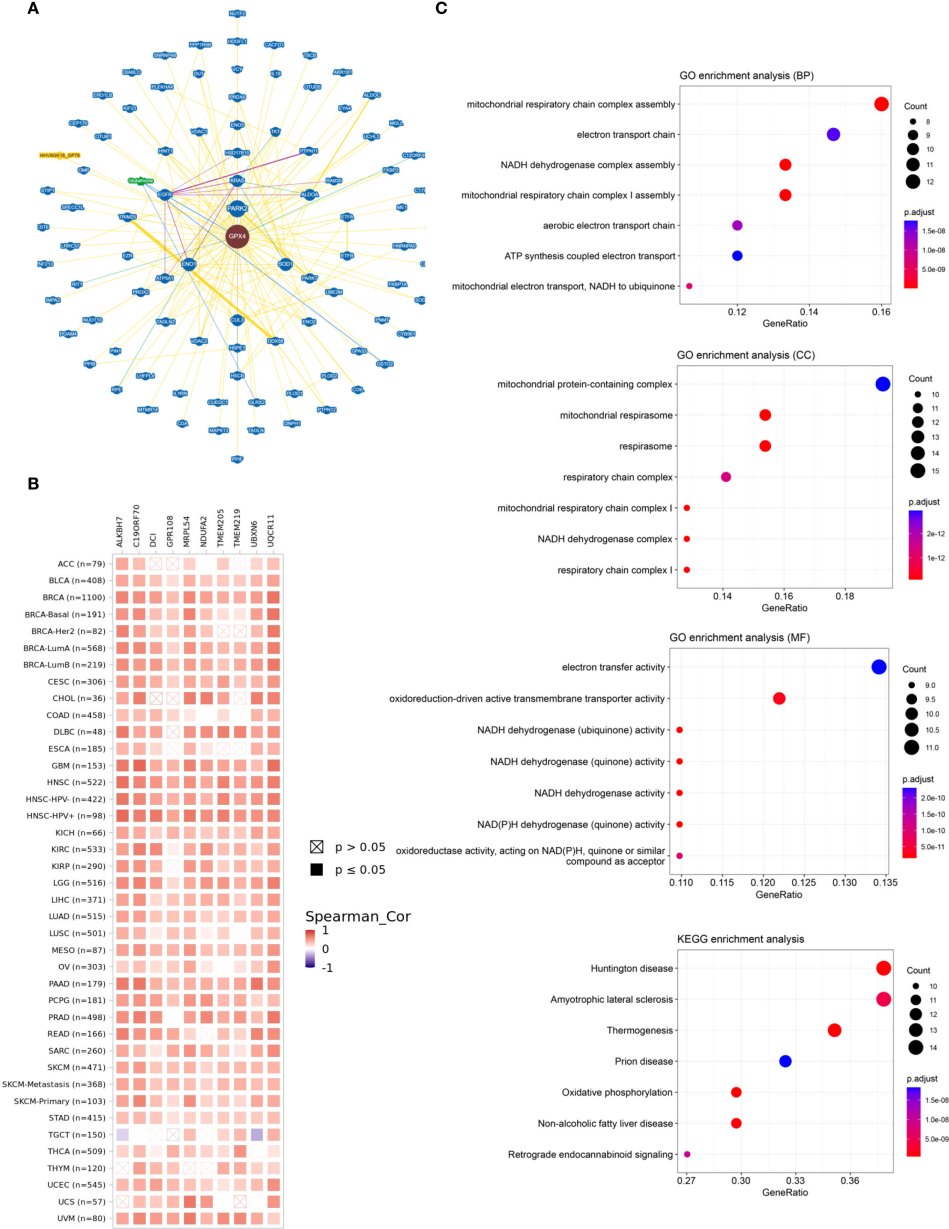
The network of protein-protein interactions and enrichment analysis of GPX4. **(A)** The interactive network of GPX4 analyzed by BioGRID; **(B)** Heat map of the correlation between GPX4 and its top 10-related genes in pan-cancer; **(C)** Gene Ontology (GO) enrichment and Kyoto Encyclopedia of Genes and Genomes (KEGG) pathway analysis based on GPX4 related genes.

Finally, we performed functional enrichment analysis of GPX4-associated genes in cancer. GPX4-interacting proteins were explored using BioGRID (**Figure 7A**). To further explore the molecular mechanisms involving GPX4, we extracted the top 100 genes associated with GPX4 from GEPIA2 (**Supplementary Table S5**). GPX4 consistently exhibited strong correlations with the top 10 most-associated genes across multiple cancer types (**Figure 7B**).

Subsequently, we carried out GO and KEGG functional enrichment analyses for GPX4. The biological processes (BP) primarily encompassed assembly of the mitochondrial respiratory chain complex, the electron transport chain, and the NADH dehydrogenase complex. The cellular components (CC) were predominantly located in complexes containing mitochondrial proteins, the mitochondrial respirasome, the respirasome itself, and the respiratory chain complex. Additionally, the enriched molecular functions (MF) pertained to electron transfer activity and the activity of oxidoreduction-driven active transmembrane transporters, aligning with its function in reducing reactive oxygen species (ROS). KEGG pathway analysis highlighted associations with Huntington’s disease, amyotrophic lateral sclerosis, thermogenesis, prion diseases, and oxidative phosphorylation (**Figure 7C**). Notably, many of these pathways and functions are linked to lipid peroxidation, underscoring the pivotal role of GPX4 in ferroptosis.

## Discussion

The high incidence and mortality rates of cancer have positioned it as a significant threat to global human health (15), prompting scientists to continuously seek out new targets and therapeutics for cancer treatment. Ferroptosis has been validated as a key player in the elimination of tumor cells and the suppression of tumor growth across numerous studies (16). GPX4 regulates ferroptosis, and its inhibition or destabilization under certain conditions can trigger ferroptosis (1). A growing body of research has confirmed that GPX4 is crucial in cancer biology, encompassing tumor viability (17), migration and invasion (18, 19), response to chemotherapy (20, 21) and modulation of the tumor immune microenvironment (22). Despite GPX4’s significant role in tumors over the past decade, the precise relationship between GPX4 and tumors remains unclear. This study’s pan-cancer analysis reveals associations between GPX4 expression and various cancer-related factors, including prognosis, DNA methylation, TMB, MSI, immunoregulatory genes, and immune cell infiltration.

The initial phase of the study concentrated on the GPX4 expression profile across various cancerous tissues, revealing that GPX4 expression was significantly higher in tumor tissues than in normal tissues for the majority of cancers examined. Functional experiments within colorectal and gastric cancer cell lines demonstrated that increased GPX4 levels boosted cellular proliferation, suggesting an oncogenic role for GPX4, a finding that aligns with previous researches (23, 24). Additionally, GPX4 expression correlated with tumor grade and cancer stage. The study’s findings suggest that GPX4 could emerge as a novel diagnostic biomarker for certain cancers, given its remarkable ability to distinguish between cancerous and healthy tissues.

Subsequently, we investigated the correlation between GPX4 expression and survival outcomes across a range of cancer types. In certain cancers, including BRCA, THCA, and UCEC, GPX4 appears to be protective, whereas in others like COAD, LAML, and UVM, it acts as a risk factor. Consequently, GPX4 could serve as a valuable tool in cancer risk prediction by offering a prognostic indicator for various types of cancer.

Furthermore, the variable expression of GPX4 in cancers might be attributed in part to the deregulation of GPX4 promoter methylation and gene mutations. The gene mutation of GPX4 did occur in certain cancers, such as SARC, ACC, Glioma, miscellaneous neuroepithelial tumors, and CHOL, OV and CESC, but the proportion of such gene mutations was no more than 8%. More research reports focus on the methylation of the GPX4 gene. In most tumors, the methylation of GPX4 was downregulated, leading to an increase in GPX4 expression and thereby exerting a carcinogenic effect, suggesting that epigenetic regulation may be involved in its aberrant overexpression. In pancreatic cancer cells, study showed that HMGA2 could bind and promote cis-element modification in the promoter region of the GPX4 gene by enhancing enhancer activity through increased H3K4 methylation and H3K27 acetylation, thus promoting GPX4 expression (25). Meng et al. (26)found that Longikaurin A inhibited ten-eleven translocation 2 (TET2), which induced GPX4 downregulation through DNA methylation, ultimately leading to ferroptosis and suppressed glioblastoma (GBM) function. This kind of epigenetic-level regulation also occurs in the regulation of inflammatory diseases (27–30).

Thanks to single cell sequencing data analysis which has the advantage of overcoming cell heterogeneity in tumors, we found that GPX4 was significantly correlated with tumor functional status, such as DNA damage, DNA repair, cell cycle, metastasis, angiogenesis, inflammation, EMT, and quiescence in some cancers. Based on these findings, GPX4 might play a crucial role in the biological processes of cancer progression.

Immunotherapy has become a prevalent method for treating cancer. The use of immune checkpoint inhibitors like PD-1, PD-L1, and CTLA-4 has enhanced the survival outcomes for certain cancer patients, particularly those with malignant melanoma, gastric carcinoma, and hepatocellular carcinoma. Despite the advantages of immunotherapy, challenges persist. Identifying new targets and biomarkers is essential for enhancing the effectiveness of immunotherapy. In this context, we explored the link between GPX4 expression and the infiltration of immune cells in tumor tissues. There is evidence suggesting that GPX4 expression levels are associated with the degree of immune cell infiltration. Studies have indicated that GPX4 was implicated in the infection by certain microorganisms, such as Mycobacterium tuberculosis (31). A recent study also demonstrated that mice deficient in GPX4 developed focal granuloma-like neutrophilic enteritis when subjected to a ω-6 polyunsaturated fatty acid (PUFA)-rich Western diet (32). Similarly, mice lacking Gpx4 exhibited more severe pancreatitis following cerulein infection or ethanol administration (33). These studies suggest that GPX4 plays a significant role in modulating immunity in certain diseases. To further assess the relationship between GPX4 and the tumor microenvironment (TME), we first analyzed the correlation between GPX4 expression and the levels of various immune cell infiltrates. A significant correlation was observed between GPX4 expression and the presence of macrophages, cancer-associated fibroblasts (CAFs), neutrophils, B cells, CD8+ T cells, and regulatory T cells (Treg) in some cancers. According to previous research, both M1 and M2 macrophages were central to inflammatory responses and tumorigenesis within the TME, with M1 macrophages primarily involved in pro-inflammatory responses and historically viewed as antitumor cells, while M2 macrophages were predominantly associated with anti-inflammatory responses and contribute to pro-tumorigenic outcomes (34). Notably, we found that GPX4 expression was positively correlated with M2 macrophages in the TME, suggesting its potential role in promoting oncogenesis in these cancers.

CAFs, a subtype of fibroblasts within the tumor microenvironment (TME) that promote cancer progression, have been extensively researched, yielding promising results that highlight their potential as therapeutic and prognostic markers (35, 36). However, treatment strategies targeting CAFs have mostly failed in clinical trials (35). The precise mechanisms involved have not yet been fully elucidated. In our current study, a positive correlation was observed between GPX4 expression and the infiltration of CAFs in ESCA, GBM, HNSC, STAD, and TGCT, indicating that GPX4 may play a role in the regulation of CAFs within the TME. This suggests that GPX4 could be a therapeutic target for modulating the tumor immune microenvironment.

Tumor-associated neutrophils (TANs) have emerged as an integral component of the TME. They serve a dual function. TANs can stimulate tumor-promoting inflammation by facilitating angiogenesis, remodeling the extracellular matrix, promoting metastasis, and suppressing the immune system. Conversely, neutrophils may also contribute to antitumor activities by directly killing tumor cells and interacting with cellular networks that combat tumor resistance (37). Interestingly, our findings showed that GPX4 expression was inversely correlated with neutrophil infiltration, particularly in CESC and HNSC. Additional research is necessary to clarify the function of GPX4 in modulating neutrophils within the TME and its implications for oncogenesis and tumor progression. Synthesizing these findings, we can infer that elevated GPX4 levels might serve as an indicator of a subdued immune response. Nevertheless, the specific mechanisms through which GPX4 influences the TME warrant further exploration.

The main constraint of our study is its heavy reliance on bioinformatics analysis as the primary research approach. While we have validated our results using cell lines, it is imperative to further substantiate our findings with larger cohorts and a more diverse range of cancer types. Using our integrated multi-omics, we discovered a crosstalk network of GPX4 in pan-cancers, providing mechanistic support for developing broad-spectrum therapies - a key advancement beyond the descriptive findings in recent reviews.

In conclusion, GPX4 shows aberrant expression patterns and is correlated with clinical outcomes and immune responses across a broad pan-cancer analysis. The function of GPX4 in tumors is not yet fully understood, and further in-depth research into its regulatory mechanisms and clinical significance will be instrumental in devising novel therapeutic strategies for the prevention and treatment of human cancers.

## Author's note

A preprint has previously been published (Xiao Li, et al. Pan-Cancer Analysis of the Role of the Ferroptosis-Related Biomarker GPX4: A Potential Target for Prognosis and Immunotherapy, 11 April 2023, PREPRINT (Version 1) available at Research Square (https://doi.org/10.21203/rs.3.rs-2762186/v1).

## Data Availability

The original contributions presented in the study are included in the article/**Supplementary Material**. Further inquiries can be directed to the corresponding author.
