# Supplementary material for: Ferroptosis’s Master Switch GPX4 emerges as universal biomarker for precision immunotherapy: a pan-cancer study with in vitro experiments validation
Source: Front Oncol. 2025 Oct 9;15:1643235. doi: 10.3389/fonc.2025.1643235 (PMC12545133; doi:10.3389/fonc.2025.1643235)
Supplement: Supplementary file 1 [file Supplementaryfile1.docx]

Supplementary Material


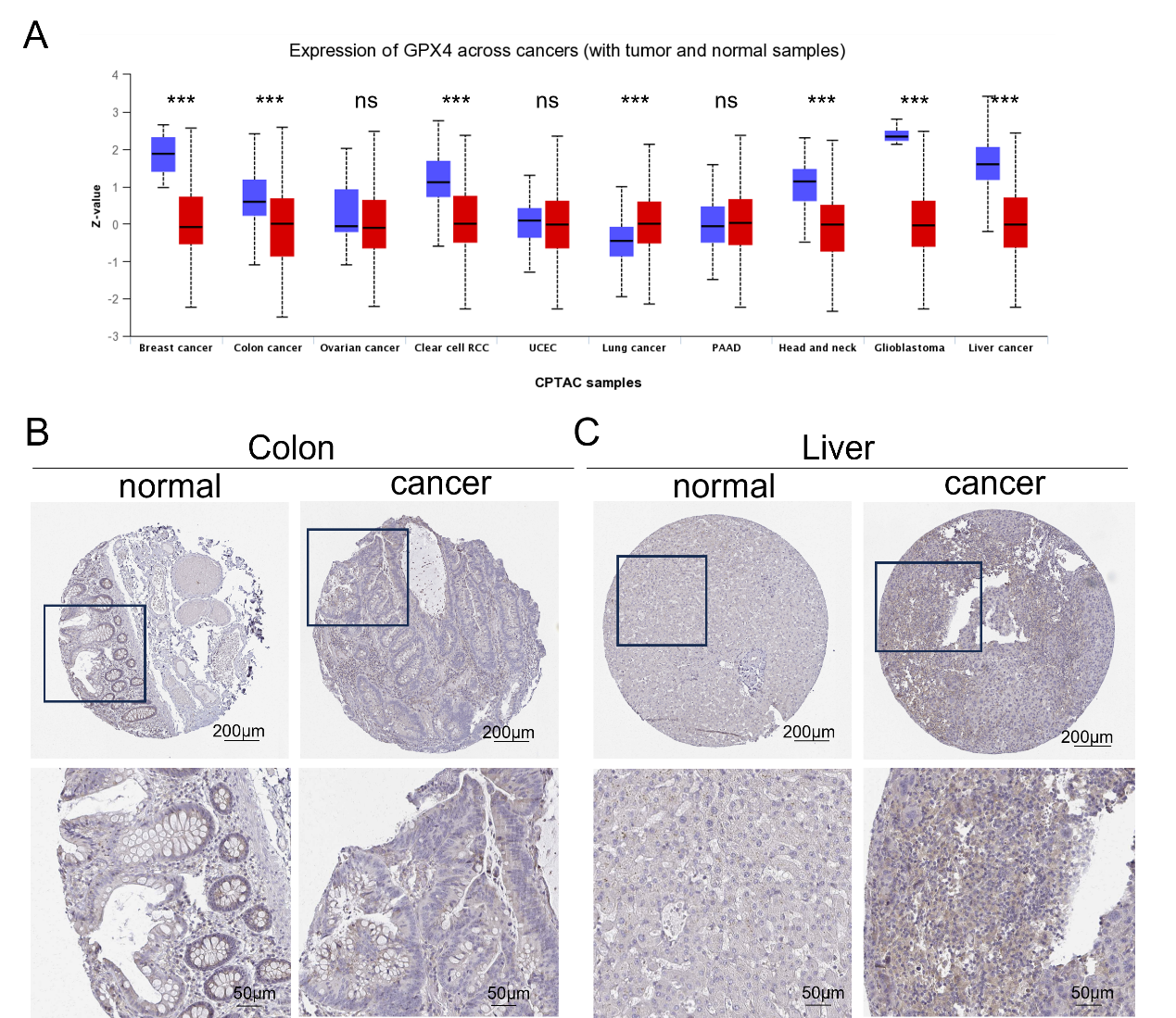


Figure S1. GPX4 expression levels in pan-cancer. (A) The protein level of GPX4 in different cancer types according to the UALCAN-CPTAC database. ns not significant. *p < 0.05; **p < 0.01; ***p < 0.001. (B-C) The results of immunohistochemistry demonstrated that the expression of GPX4 in colon cancer and liver cancer was significantly higher than that in the corresponding normal tissues. (The data is sourced from the The Human Protein Atlas)


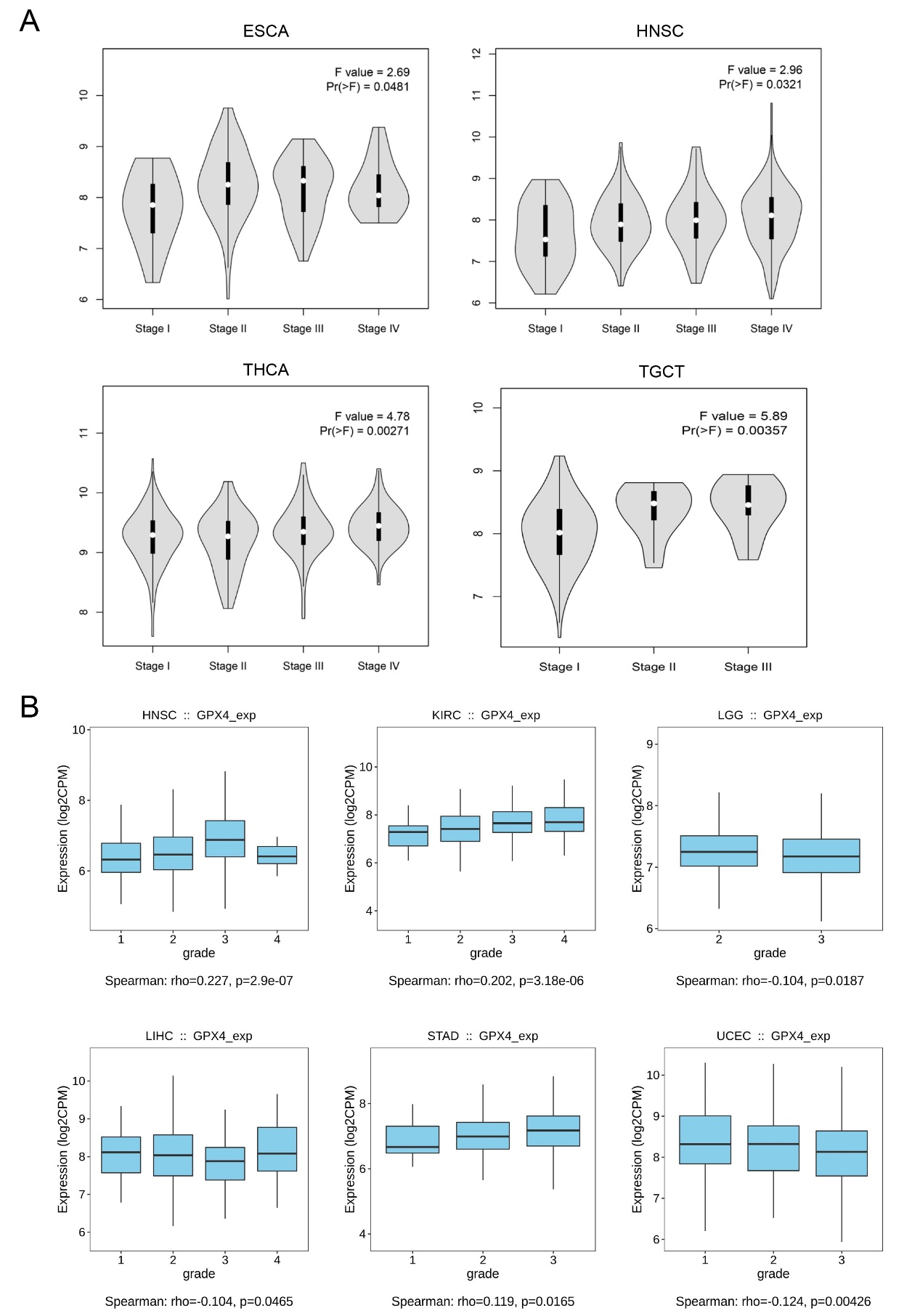


Figure S2. Relationship between GPX4 expression levels and tumor pathological stages and tumor grade in different cancer. (A) Relationship between GPX4 expression levels and tumor pathological stages in different cancer performed in GEPIA2. (B) Relationship between GPX4 expression levels and tumor grade analyzed by TISIDB.


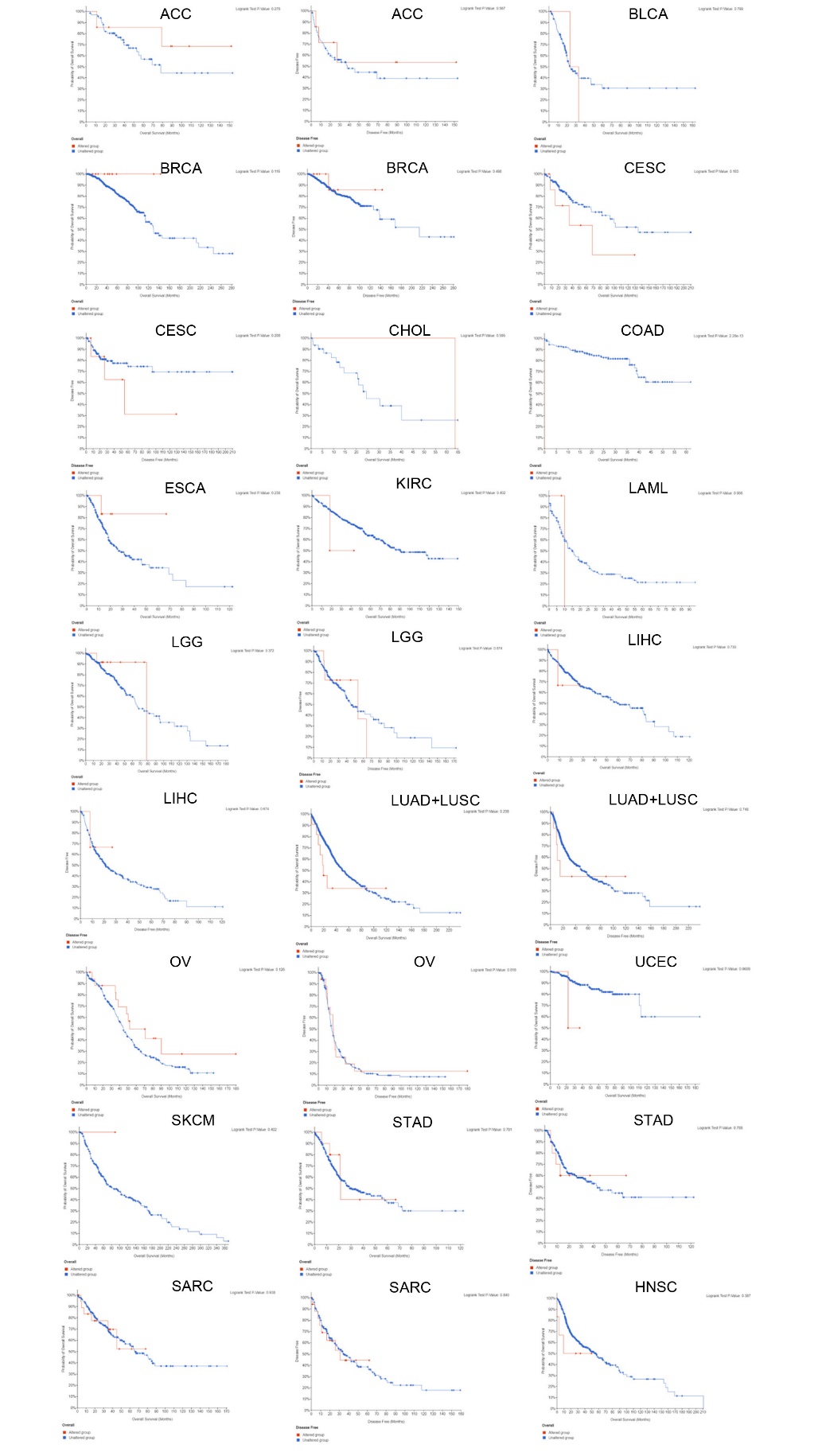


Figure S3. Roles of GPX4 alteration in the patients’ prognosis in various cancers.


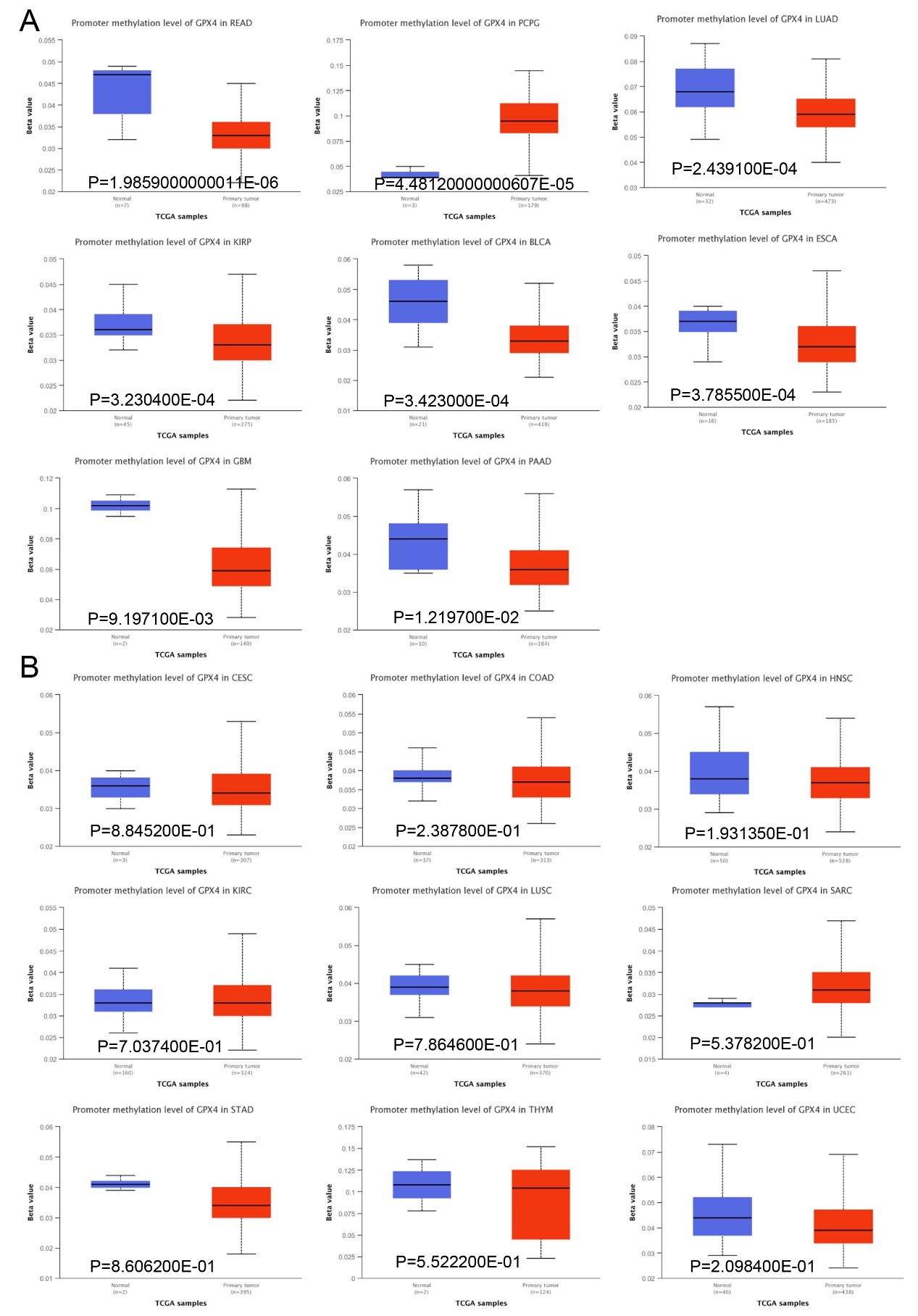


Figure S4. Methylation levels of GPX4 in various cancers. (A) Cancers with significant differences and (B) cancers with non-significant differences in GPX4 methylation levels when comparing cancerous and normal tissues.


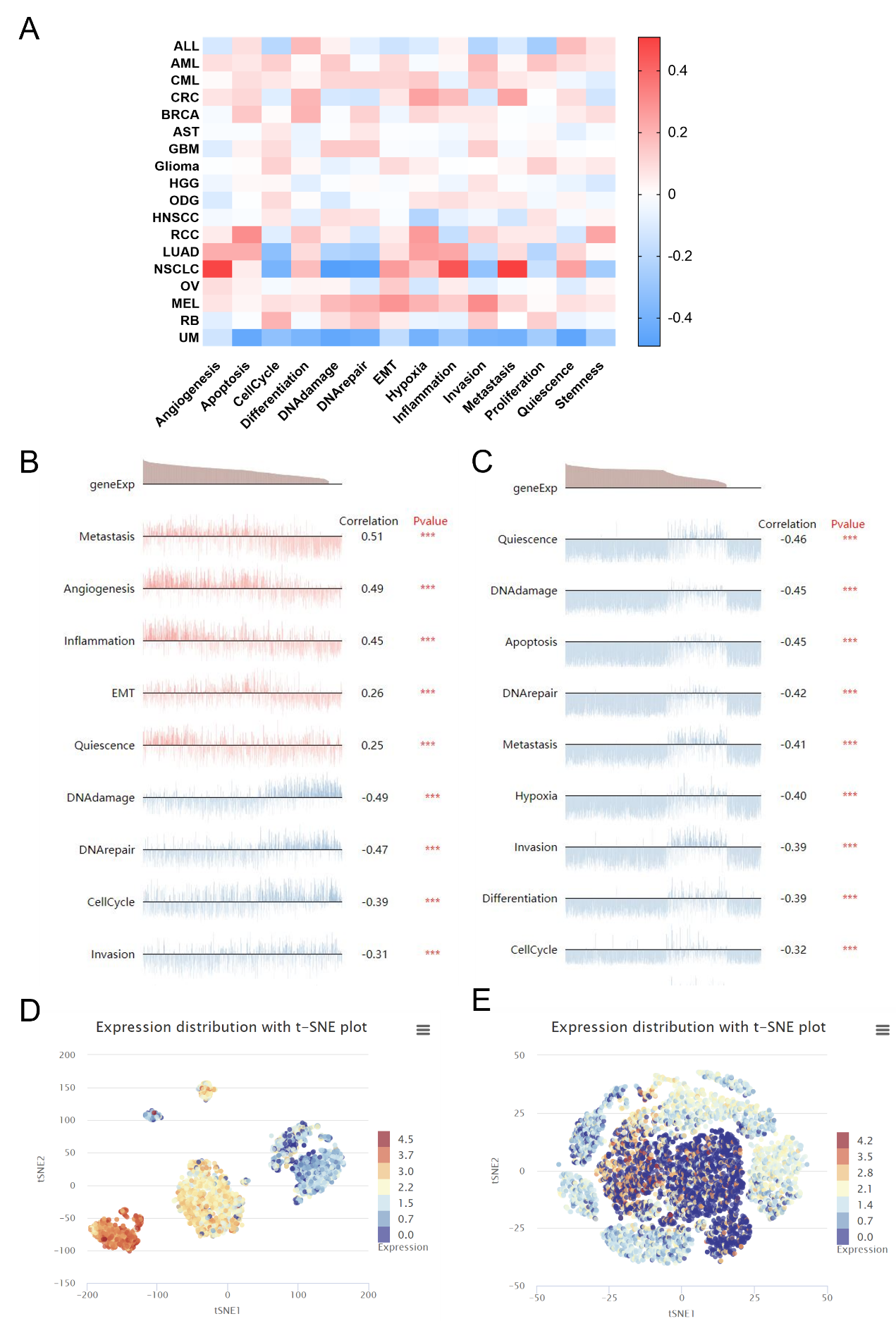


**Figure S5:** The expression pattern of GPX4 at the single-cell level. (A) A heatmap displaying the correlation between GPX4 expression and different tumor functional status was performed by the CancerSEA database; (B, C) Correlation between GPX4 expression and tumor function in NSCLE (B), and UM (C) samples. ***p < 0.001; (D-E) The t-SNE diagrams portrayed the distributions of GPX4 at single-cell levels in NSCLE (D) and UM (E) samples. T-SNE describes the distribution of cells, every point represents a single cell, and the color of the point represents the expression level of the gene in the cell.


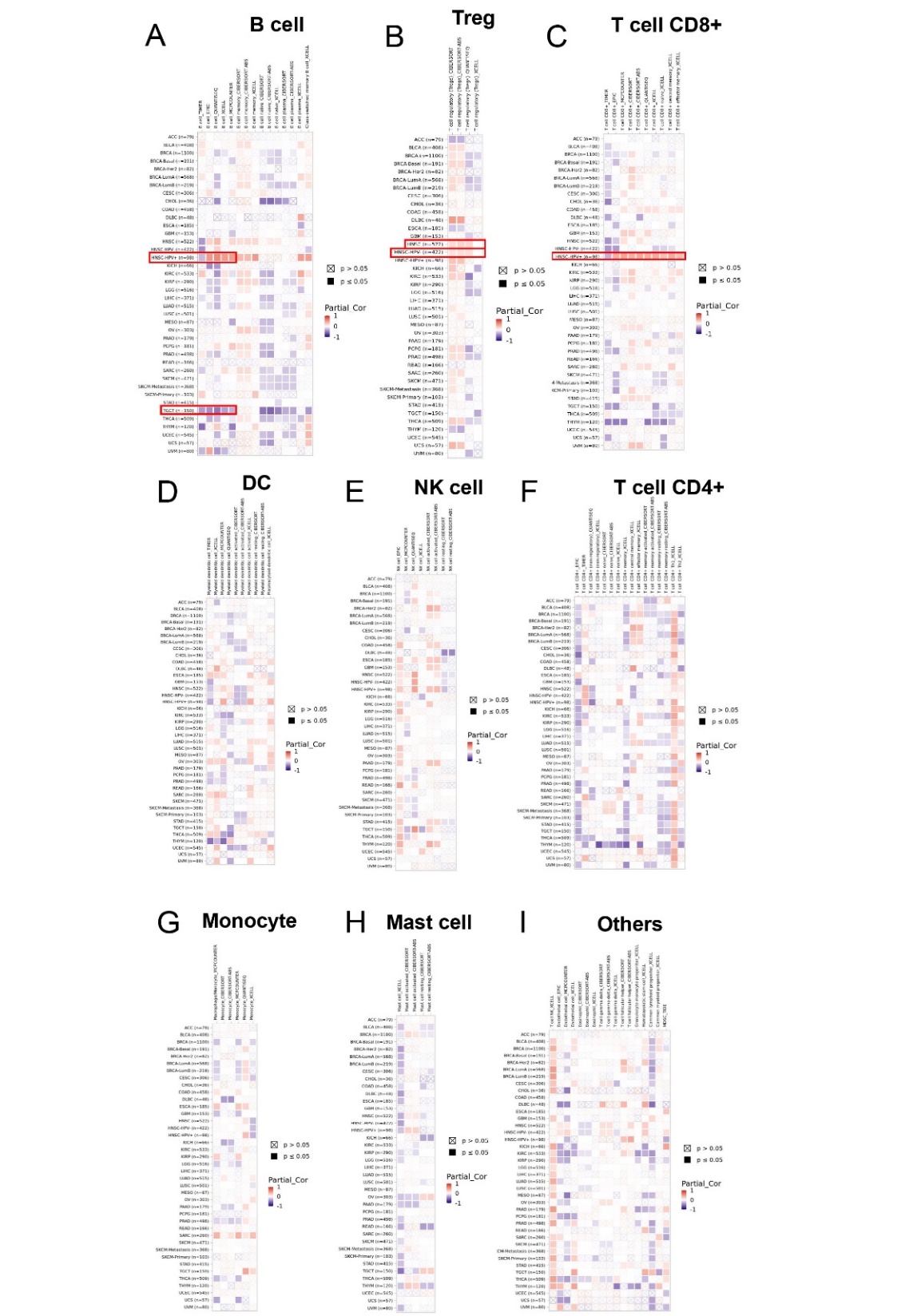


Figure S6. Correlation heatmap between GPX4 expression and tumor infiltrating immune cells across 33 cancer types was displayed. The TIMER2.0 database depicted the relationship between GPX4 expression and immune infiltration of B cell (A), Treg (B), T cell CD8+ (C), dendritic cells(D), natural killer cells(E), CD4+ T cells(F), monocytes(G), Mast cell(H) and other immune cell types(I) through several algorithms.


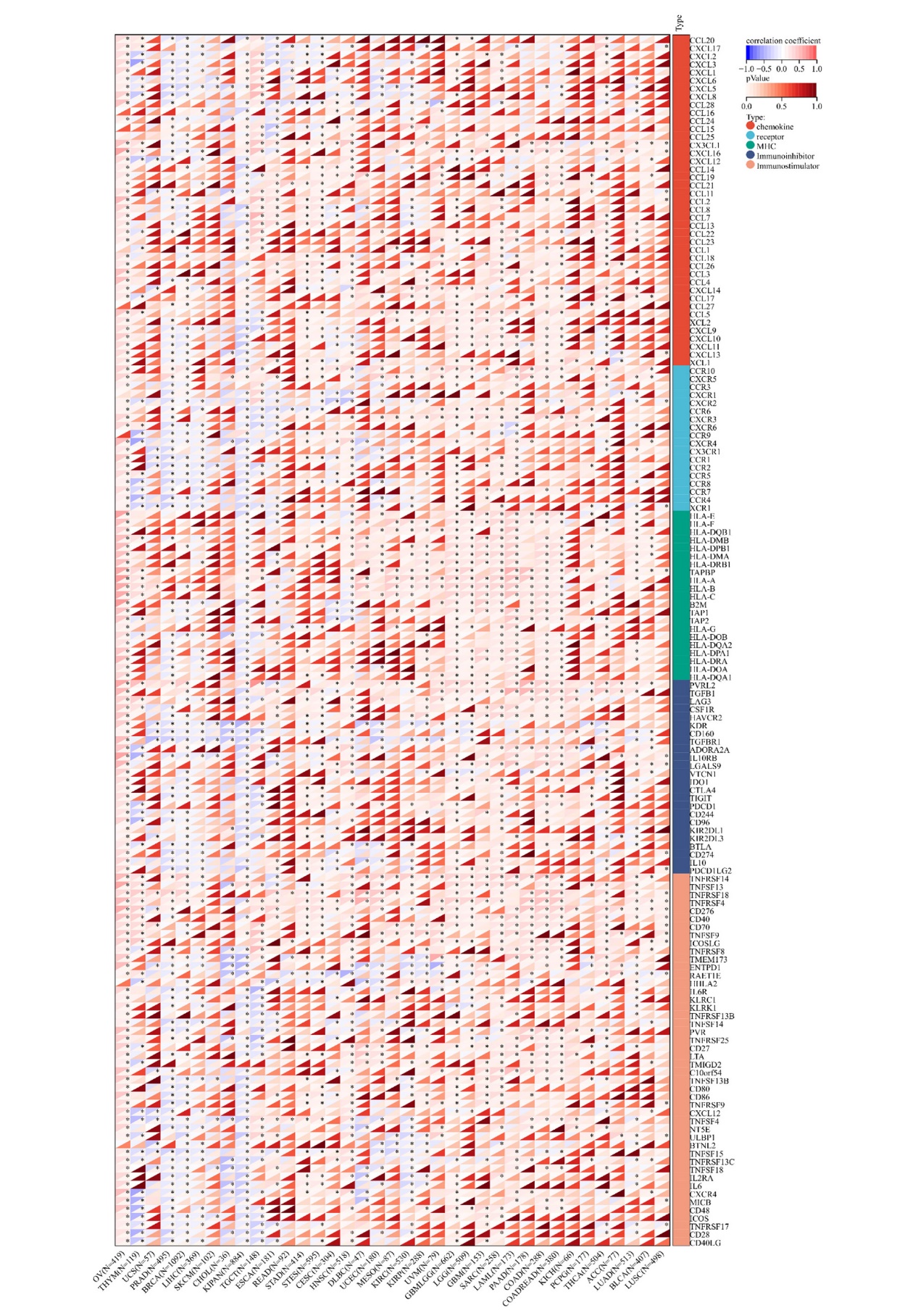


Figure S7 Correlation heatmap of GPX4 expression with immune-related genes across different cancer types. *Statistically significant.


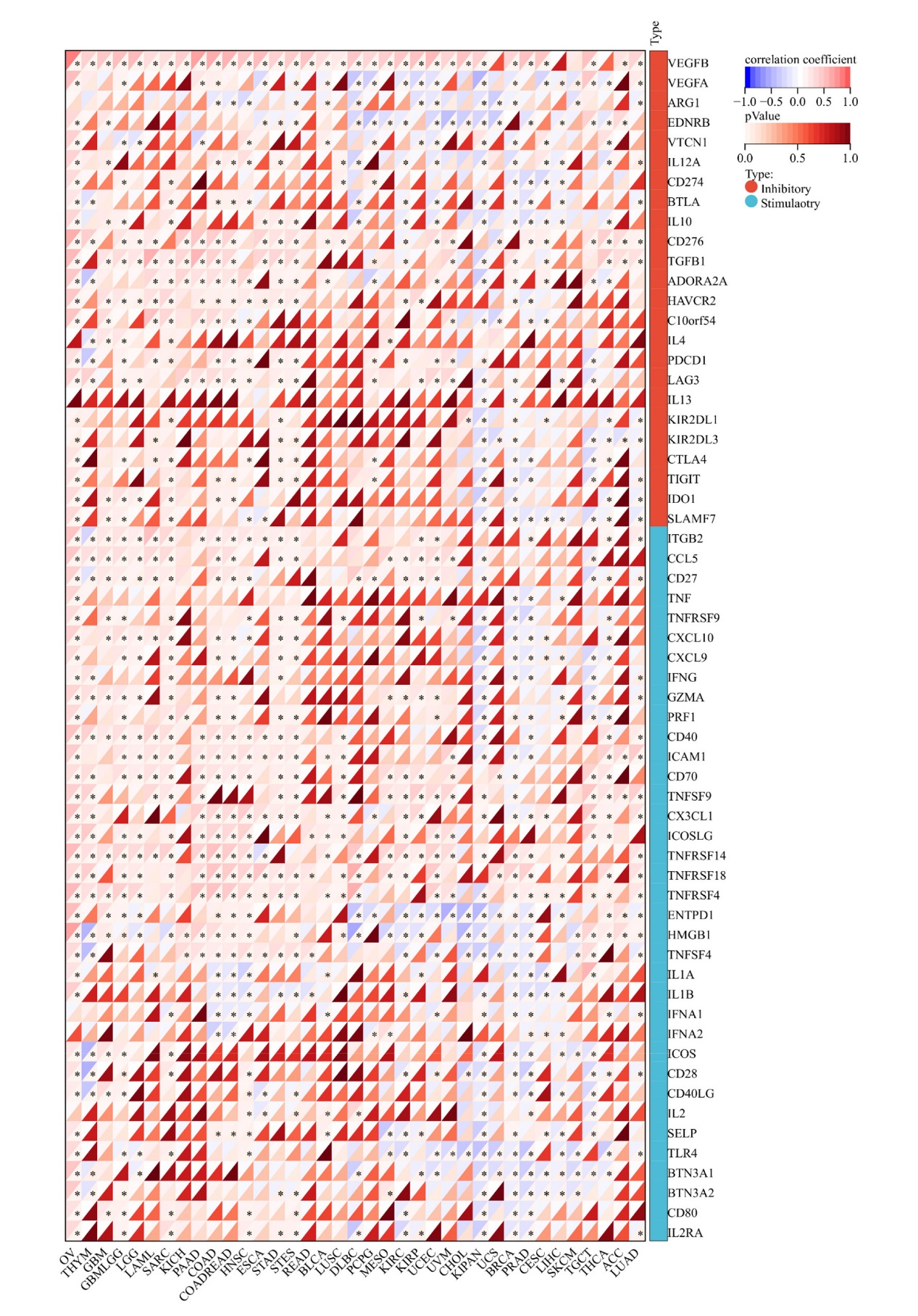


Figure S8. Correlation between GPX4 and immune checkpoint genes across cancer types. *Statistically significant. *p < 0.05, **p < 0.01, ***p < 0.001.


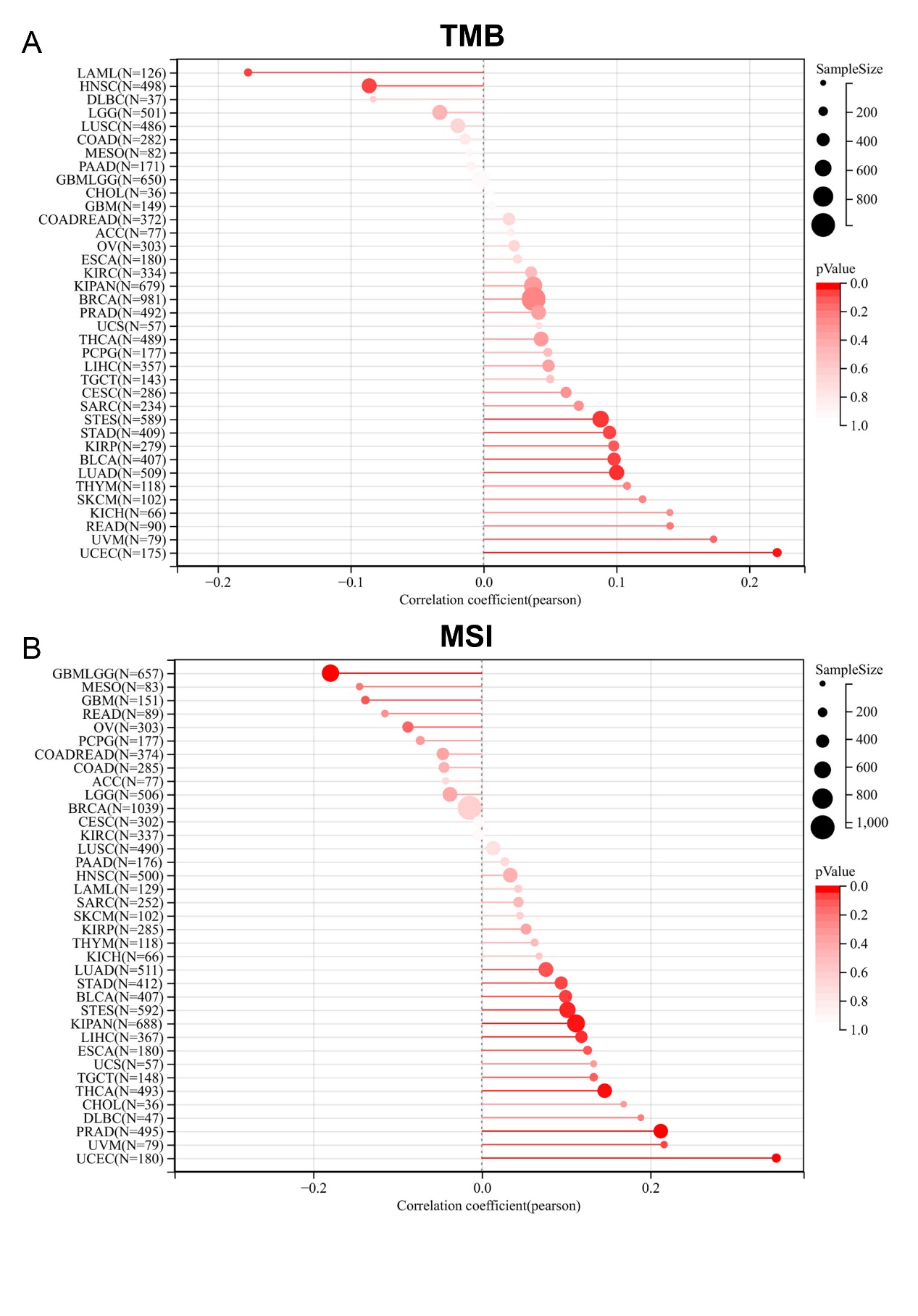


Figure S9. The relationship between GPX4 expression and tumor mutation burden (TMB) (**A**) and microsatellite instability (MSI) (B).


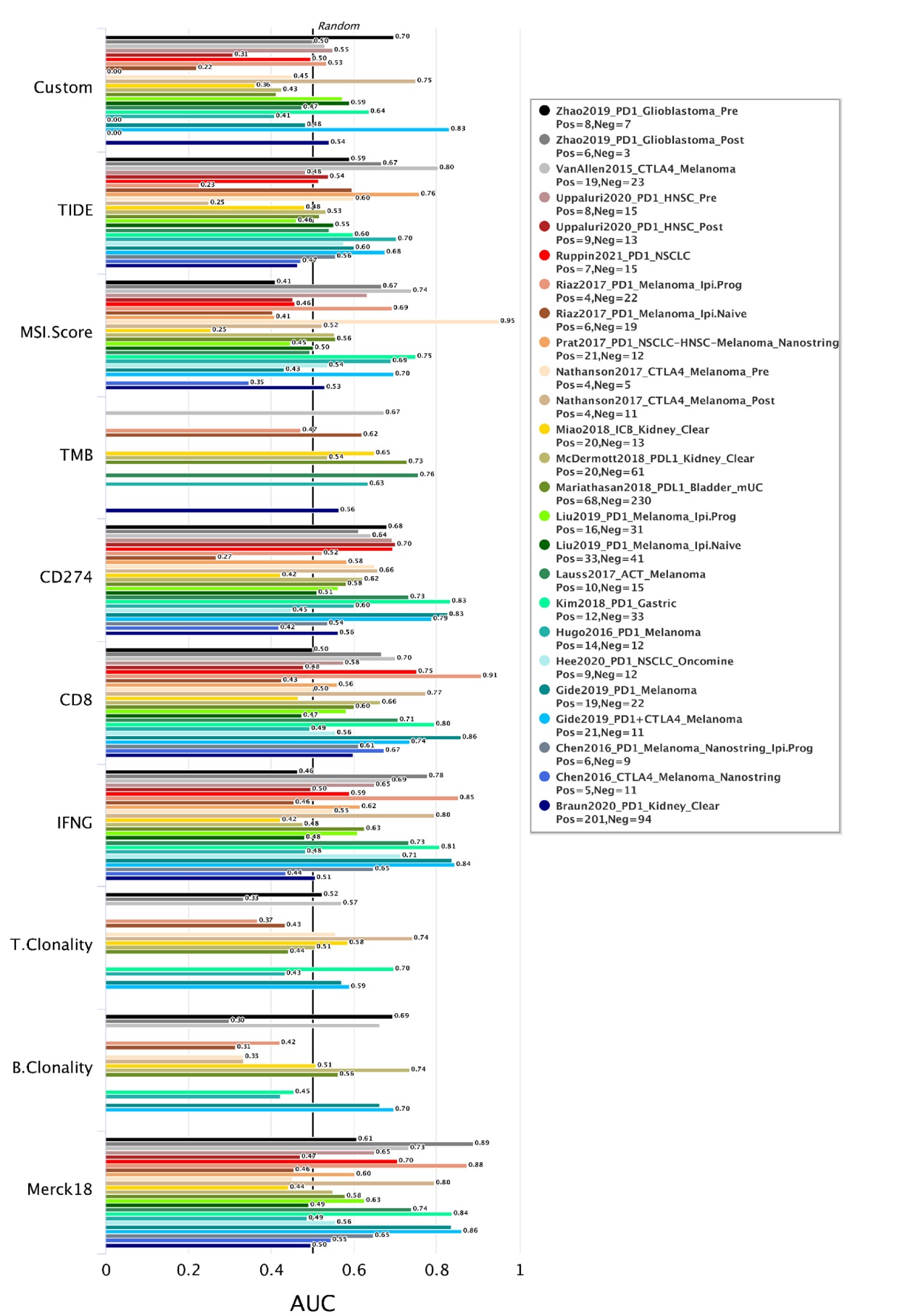


Figure S10. The ability of GPX4 in predicting response to immunotherapy


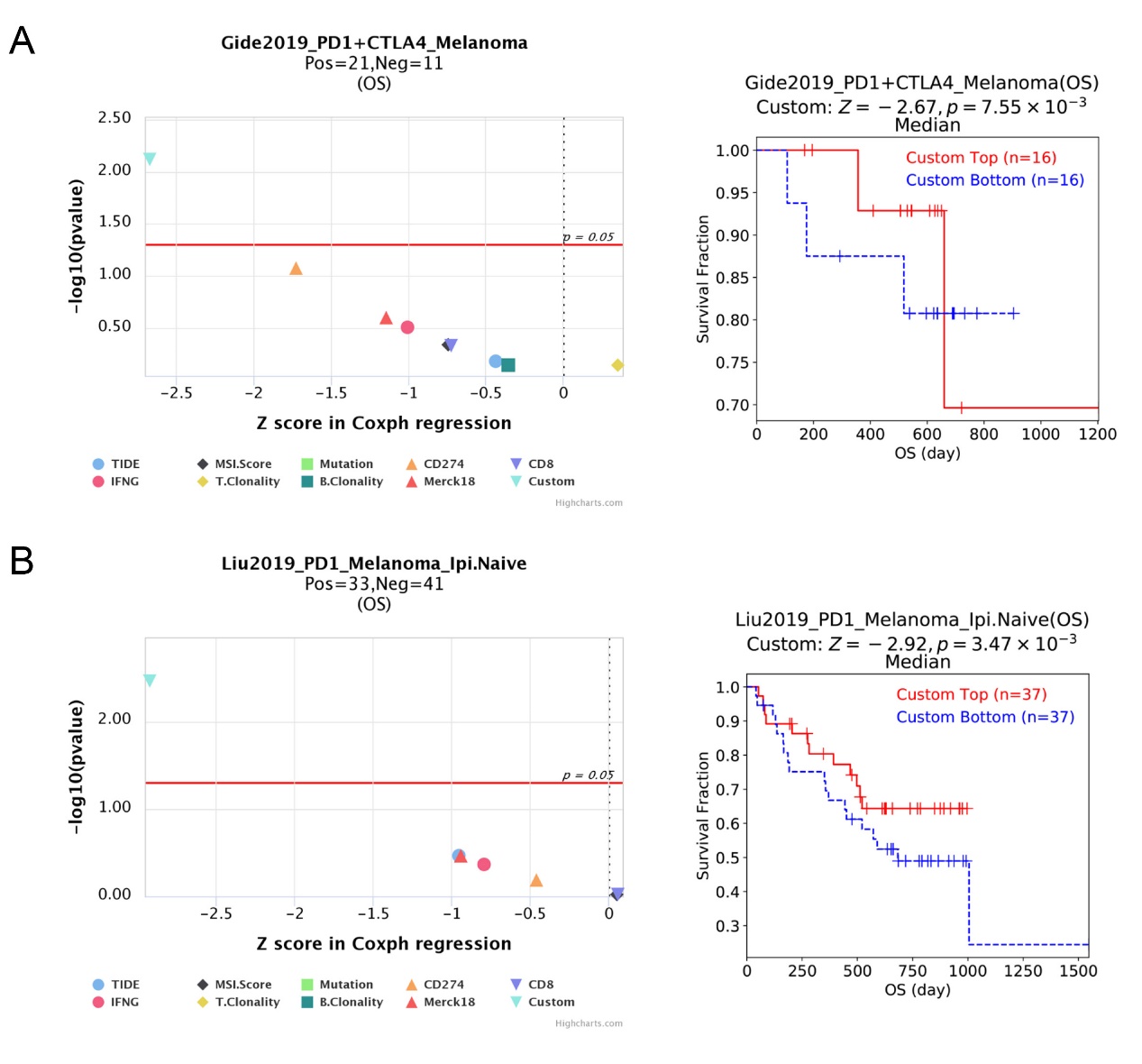


Figure S11 Association between GPX4 expression and biotherapy outcome in Gide2019 (A) and Liu2019 (B) clinical studies of immune checkpoint blockade.
