# Supplementary material for: Ferroptosis’s Master Switch GPX4 emerges as universal biomarker for precision immunotherapy: a pan-cancer study with in vitro experiments validation
Source: Front Oncol. 2025 Oct 9;15:1643235. doi: 10.3389/fonc.2025.1643235 (PMC12545133; doi:10.3389/fonc.2025.1643235)
Supplement: Supplementary file 2 [file Table1.docx]

Supplementary Table S1. The value of GPX4 in differentiating cancer from normal tissues in various cancer based on TCGA datasets.

| Cancer type | Tumor | Normal | AUC | 95%CI |
| --- | --- | --- | --- | --- |
| THCA | 510 | 58 | 0.868 | 0.826-0.911 |
| KIRP | 289 | 32 | 0.864 | 0.814-0.914 |
| UCEC | 181 | 406 | 0.859 | 0.815-0.902 |
| READ | 167 | 10 | 0.854 | 0.777-0.932 |
| LIHC | 374 | 50 | 0.838 | 0.791-0.885 |
| ESCA | 162 | 11 | 0.822 | 0.717-0.926 |
| COAD | 480 | 41 | 0.815 | 0.760-0.870 |
| KIRC | 539 | 72 | 0.759 | 0.711-0.808 |
| CHOL | 36 | 9 | 0.753 | 0.601-0.905 |
| HNSC | 502 | 44 | 0.655 | 0.570-0.739 |
| PRAD | 499 | 52 | 0.647 | 0.572-0.723 |
| LUAD | 535 | 59 | 0.637 | 0.586-0.689 |
| STAD | 375 | 32 | 0.637 | 0.548−0.726 |
| BLCA | 414 | 19 | 0.632 | 0.519-0.746 |
| BRCA | 1109 | 113 | 0.571 | 0.514-0.629 |
| KICH | 65 | 24 | 0.554 | 0.416-0.692 |
| LUSC | 502 | 49 | 0.525 | 0.466-0.585 |

AUC, area under the curve; CI, confidence interval; THCA, thyroid carcinoma; KIRP, kidney renal papillary cell carcinoma; UCEC, uterine corpus endometrial carcinoma; READ, rectum adenocarcinoma; LIHC, liver hepatocellular carcinoma; ESCA, esophageal carcinoma; COAD, colon adenocarcinoma; KIRC, kidney renal clear cell carcinoma; CHOL, cholangio carcinoma; HNSC, head and neck squamous cell carcinoma; PRAD, prostate adenocarcinoma; LUAD, lung adenocarcinoma; STAD, stomach adenocarcinoma; BLCA, bladder urothelial carcinoma; BRCA, breast invasive carcinoma; KICH, kidney chromophobe; LUSC, lung squamous cell carcinoma;
