# Supplementary material for: Ferroptosis’s Master Switch GPX4 emerges as universal biomarker for precision immunotherapy: a pan-cancer study with in vitro experiments validation
Source: Front Oncol. 2025 Oct 9;15:1643235. doi: 10.3389/fonc.2025.1643235 (PMC12545133; doi:10.3389/fonc.2025.1643235)
Supplement: Supplementary file 3 [file Table2.docx]

Supplementary Table S2. The value of GPX4 in differentiating cancer from normal tissues in various cancer based on TCGA and GTEx datasets.

| Cancer type | Tumor | Normal | AUC | 95%CI |
| --- | --- | --- | --- | --- |
| PAAD | 179 | 171 | 0.979 | 0.963-0.996 |
| LAML | 173 | 70 | 0.976 | 0.954-0.997 |
| THYM | 119 | 446 | 0.970 | 0.958-0.983 |
| TGCT | 154 | 165 | 0.950 | 0.923-0.976 |
| DLBC | 47 | 444 | 0.940 | 0.913-0.966 |
| THCA | 512 | 338 | 0.930 | 0.913-0.947 |
| LIHC | 371 | 160 | 0.904 | 0.879-0.930 |
| KIRP | 289 | 60 | 0.890 | 0.850-0.929 |
| GBM | 166 | 1157 | 0.887 | 0.859-0.915 |
| OV | 427 | 88 | 0.877 | 0.845-0.908 |
| STAD | 414 | 210 | 0.841 | 0.807-0.874 |
| UCEC | 552 | 35 | 0.835 | 0.783-0.887 |
| LGG | 523 | 1152 | 0.830 | 0.810-0.849 |
| UCS | 57 | 78 | 0.808 | 0.734-0.882 |
| KICH | 66 | 53 | 0.754 | 0.663-0.845 |
| PRAD | 496 | 152 | 0.735 | 0.692-0.778 |
| ESCA | 182 | 666 | 0.729 | 0.683-0.775 |
| COAD | 290 | 349 | 0.720 | 0.680-0.760 |
| ACC | 77 | 128 | 0.718 | 0.638-0.798 |
| KIRC | 531 | 100 | 0.713 | 0.664-0.762 |
| READ | 93 | 318 | 0.707 | 0.646-0.767 |
| LUAD | 515 | 347 | 0.698 | 0.663-0.732 |
| SKCM | 469 | 813 | 0.686 | 0.656-0.717 |
| BRCA | 1099 | 292 | 0.645 | 0.608-0.682 |
| BLCA | 28 | 407 | 0.590 | 0.498-0.681 |
| LUSC | 498 | 338 | 0.575 | 0.537-0.614 |
| CESC | 306 | 13 | 0.562 | 0.407-0.717 |

AUC, area under the curve; CI, confidence interval; PAAD, pancreatic adenocarcinoma; LAML, acute myeloid leukemia; THYM, thymoma; TGCT, testicular germ cell tumors; DLBC, diffuse large B-cell lymphoma; THCA, thyroid carcinoma; LIHC, liver hepatocellular carcinoma; KIRP, kidney renal papillary cell carcinoma; GBM, glioblastoma multiforme; OV, ovarian serous cystadenocarcinoma; STAD, stomach adenocarcinoma; UCEC, uterine corpus endometrial carcinoma; LGG, brain lower grade glioma; UCS, uterine carcinosarcoma; KICH, kidney chromophobe; PRAD, prostate adenocarcinoma; ESCA, esophageal carcinoma; COAD, colon adenocarcinoma; ACC, adrenocortical carcinoma; KIRC, kidney renal clear cell carcinoma; READ, rectum adenocarcinoma; LUAD, lung adenocarcinoma; SKCM, skin cutaneous melanoma; BRCA, breast invasive carcinoma; BLCA, bladder urothelial carcinoma; LUSC, lung squamous cell carcinoma; CESC, cervical squamous cell carcinoma and endocervical adenocarcinoma;
