# Supplementary material for: Ferroptosis’s Master Switch GPX4 emerges as universal biomarker for precision immunotherapy: a pan-cancer study with in vitro experiments validation
Source: Front Oncol. 2025 Oct 9;15:1643235. doi: 10.3389/fonc.2025.1643235 (PMC12545133; doi:10.3389/fonc.2025.1643235)
Supplement: Supplementary file 4 [file Table3.docx]

Supplementary Table S3. Correlations between GPX4 expression and disease stage

| Cancer type | F value | Pr(>F) |
| --- | --- | --- |
| ACC | 0.0972 | 0.961 |
| BLCA | 0.0192 | 0.981 |
| BRCA | 1.02 | 0.395 |
| CESC | 0.47 | 0.704 |
| CHOL | 1.52 | 0.233 |
| COAD | 0.0584 | 0.981 |
| DLBC | 0.28 | 0.84 |
| ESCA | 2.69 | 0.0481 |
| HNSC | 2.96 | 0.0321 |
| KICH | 2.11 | 0.108 |
| KIRC | 1.94 | 0.122 |
| KIRP | 1.2 | 0.309 |
| LIHC | 0.42 | 0.739 |
| LUAD | 0.226 | 0.878 |
| LUSC | 1.15 | 0.329 |
| OV | 0.447 | 0.64 |
| PAAD | 2.06 | 0.108 |
| READ | 0.273 | 0.845 |
| SKCM | 1.05 | 0.382 |
| STAD | 1.34 | 0.26 |
| TGCT | 5.89 | 0.00357 |
| THCA | 4.78 | 0.00271 |
| UCEC | 1.23 | 0.301 |
| UCS | 1.37 | 0.261 |

ACC, adrenocortical carcinoma; BLCA, bladder urothelial carcinoma; BRCA, breast invasive carcinoma; CESC, cervical squamous cell carcinoma and endocervical adenocarcinoma; CHOL, cholangio carcinoma; COAD, colon adenocarcinoma; DLBC, diffuse large B-cell lymphoma; ESCA, esophageal carcinoma; GBM, glioblastoma multiforme; HNSC, head and neck squamous cell carcinoma; KICH, kidney chromophobe; KIRC, kidney renal clear cell carcinoma; KIRP, kidney renal papillary cell carcinoma; LIHC, liver hepatocellular carcinoma; LUAD, lung adenocarcinoma; LUSC, lung squamous cell carcinoma; OV, ovarian serous cystadenocarcinoma; PAAD, pancreatic adenocarcinoma; READ, rectum adenocarcinoma; SKCM, skin cutaneous melanoma; STAD, stomach adenocarcinoma; TGCT, testicular germ cell tumors; THCA, thyroid carcinoma; UCEC, uterine corpus endometrial carcinoma; UCS, uterine carcinosarcoma.
