# Supplementary material for: Ferroptosis’s Master Switch GPX4 emerges as universal biomarker for precision immunotherapy: a pan-cancer study with in vitro experiments validation
Source: Front Oncol. 2025 Oct 9;15:1643235. doi: 10.3389/fonc.2025.1643235 (PMC12545133; doi:10.3389/fonc.2025.1643235)
Supplement: Supplementary file 5 [file Table4.docx]

Supplementary table S4. GPX4 expression and immune infiltration scores in pan-cancer.

| Cancer type | StromalScore | | ImmuneScore | | ESTIMATEScore | |
| --- | --- | --- | --- | --- | --- | --- |
|  | R | P | R | P | R | P |
| TCGA-GBM(N=152) | 0.037301 | 0.648216 | 0.145563 | 0.073559 | 0.103209 | 0.205755 |
| TCGA-GBMLGG(N=656) | 0.183437 | 2.25E-06 | 0.19584 | 4.29E-07 | 0.195739 | 4.35E-07 |
| TCGA-LGG(N=504) | 0.10895 | 0.014399 | 0.107344 | 0.015916 | 0.111046 | 0.012614 |
| TCGA-CESC(N=291) | -0.00571 | 0.922775 | -0.10711 | 0.068078 | -0.0723 | 0.218797 |
| TCGA-LUAD(N=500) | -0.08356 | 0.061899 | -0.03786 | 0.398261 | -0.06342 | 0.156782 |
| TCGA-COAD(N=282) | 0.055415 | 0.353843 | 0.058098 | 0.330989 | 0.059933 | 0.315924 |
| TCGA-COADREAD(N=373) | 0.090332 | 0.081454 | 0.055794 | 0.282471 | 0.078612 | 0.129648 |
| TCGA-LAML(N=149) | 0.055564 | 0.500911 | 0.225984 | 0.005585 | 0.167588 | 0.041061 |
| TCGA-BRCA(N=1077) | -0.04526 | 0.137743 | -0.08207 | 0.007041 | -0.07421 | 0.014859 |
| TCGA-ESCA(N=181) | 0.187179 | 0.011632 | -0.0458 | 0.540395 | 0.075383 | 0.313174 |
| TCGA-STES(N=569) | 0.192017 | 3.96E-06 | 0.04018 | 0.338714 | 0.124062 | 0.003034 |
| TCGA-SARC(N=258) | 0.176954 | 0.004358 | 0.302206 | 7.55E-07 | 0.271965 | 9.38E-06 |
| TCGA-KIRP(N=285) | 0.005538 | 0.925838 | 0.180861 | 0.002175 | 0.117707 | 0.047111 |
| TCGA-KIPAN(N=878) | -0.47748 | 3.39E-51 | -0.24466 | 1.96E-13 | -0.37425 | 1.41E-30 |
| TCGA-STAD(N=388) | 0.181735 | 0.00032 | 0.050821 | 0.318048 | 0.126029 | 0.012978 |
| TCGA-PRAD(N=495) | -0.10142 | 0.024037 | -0.04829 | 0.283569 | -0.07846 | 0.081179 |
| TCGA-UCEC(N=178) | -0.12111 | 0.10732 | 0.163279 | 0.029428 | 0.057523 | 0.445659 |
| TCGA-HNSC(N=517) | 0.091507 | 0.037527 | 0.048713 | 0.268899 | 0.077691 | 0.077581 |
| TCGA-KIRC(N=528) | -0.28665 | 1.91E-11 | -0.03871 | 0.374636 | -0.16249 | 0.000177 |
| TCGA-LUSC(N=491) | -0.06393 | 0.157244 | -0.03927 | 0.385189 | -0.05385 | 0.233587 |
| TCGA-THYM(N=118) | 0.027484 | 0.767668 | -0.31396 | 0.000536 | -0.21849 | 0.017459 |
| TCGA-LIHC(N=363) | -0.13716 | 0.008882 | -0.13204 | 0.011802 | -0.14612 | 0.00528 |
| TCGA-THCA(N=503) | -0.04009 | 0.369552 | -0.02587 | 0.562757 | -0.03352 | 0.453218 |
| TCGA-MESO(N=85) | -0.03599 | 0.743662 | 0.21077 | 0.052838 | 0.128878 | 0.239789 |
| TCGA-READ(N=91) | 0.240881 | 0.021444 | 0.046504 | 0.661589 | 0.163608 | 0.121237 |
| TCGA-SKCM-M(N=351) | 0.073117 | 0.171691 | 0.083517 | 0.118326 | 0.085695 | 0.108997 |
| TCGA-SKCM(N=452) | 0.053365 | 0.257544 | 0.076258 | 0.105419 | 0.072846 | 0.121985 |
| TCGA-PAAD(N=177) | 0.016685 | 0.825541 | -0.02812 | 0.71022 | -0.00641 | 0.932514 |
| TCGA-OV(N=417) | -0.00693 | 0.887778 | 0.099075 | 0.04317 | 0.055374 | 0.25922 |
| TCGA-TGCT(N=132) | 0.29282 | 0.000656 | -0.12974 | 0.138164 | 0.021785 | 0.804179 |
| TCGA-PCPG(N=177) | -0.19907 | 0.007901 | 0.016707 | 0.825319 | -0.09815 | 0.193703 |
| TCGA-SKCM-P(N=101) | 0.010791 | 0.914707 | 0.100586 | 0.316907 | 0.073672 | 0.46406 |
| TCGA-UVM(N=79) | 0.342108 | 0.002029 | 0.197956 | 0.080329 | 0.254768 | 0.02346 |
| TCGA-UCS(N=56) | -0.233 | 0.083959 | 0.155604 | 0.252143 | -0.02676 | 0.844804 |
| TCGA-BLCA(N=405) | 0.080554 | 0.105504 | 0.058493 | 0.240185 | 0.074155 | 0.136282 |
| TCGA-ACC(N=77) | 0.041014 | 0.723219 | -0.13156 | 0.25407 | -0.06181 | 0.593331 |
| TCGA-KICH(N=65) | -0.03493 | 0.782394 | -0.04323 | 0.732421 | -0.04116 | 0.744747 |
| TCGA-CHOL(N=36) | -0.13058 | 0.447789 | -0.06173 | 0.720612 | -0.09261 | 0.591136 |
| TCGA-DLBC(N=46) | 0.070008 | 0.643853 | 0.102796 | 0.496622 | 0.103656 | 0.49301 |
