# Supplementary material for: Ferroptosis’s Master Switch GPX4 emerges as universal biomarker for precision immunotherapy: a pan-cancer study with in vitro experiments validation
Source: Front Oncol. 2025 Oct 9;15:1643235. doi: 10.3389/fonc.2025.1643235 (PMC12545133; doi:10.3389/fonc.2025.1643235)
Supplement: Supplementary file 6 [file Table5.docx]

Supplementary table S5. Top 100 GPX4-associated genes from GEPIA2.

| Gene Symbol | Gene ID | PCC | Gene Symbol | Gene ID | PCC |
| --- | --- | --- | --- | --- | --- |
| UBXN6 | ENSG00000167671.11 | 0.49 | WDR13 | ENSG00000101940.17 | 0.37 |
| C19orf70 | ENSG00000174917.8 | 0.47 | MIEF2 | ENSG00000177427.12 | 0.37 |
| TMEM205 | ENSG00000105518.13 | 0.47 | MRPL43 | ENSG00000055950.16 | 0.37 |
| ECI1 | ENSG00000167969.12 | 0.47 | RP11-266L9.8 | ENSG00000275494.1 | 0.37 |
| MRPL54 | ENSG00000183617.4 | 0.46 | NUDT16L1 | ENSG00000168101.14 | 0.37 |
| NDUFA2 | ENSG00000131495.8 | 0.46 | CCDC106 | ENSG00000173581.7 | 0.37 |
| GPR108 | ENSG00000125734.13 | 0.45 | ACBD4 | ENSG00000181513.14 | 0.37 |
| TMEM219 | ENSG00000149932.16 | 0.44 | ZNHIT1 | ENSG00000106400.11 | 0.36 |
| UQCR11 | ENSG00000127540.11 | 0.44 | IFT43 | ENSG00000119650.12 | 0.36 |
| ALKBH7 | ENSG00000125652.7 | 0.44 | HMGCL | ENSG00000117305.14 | 0.36 |
| NDUFA13 | ENSG00000186010.18 | 0.43 | HYI | ENSG00000178922.16 | 0.36 |
| NDUFS7 | ENSG00000115286.19 | 0.43 | MRPL53 | ENSG00000204822.6 | 0.36 |
| POLR2E | ENSG00000099817.11 | 0.43 | WDR83OS | ENSG00000105583.9 | 0.36 |
| NDUFA11 | ENSG00000174886.12 | 0.43 | RNASEK | ENSG00000219200.10 | 0.36 |
| C19orf24 | ENSG00000228300.13 | 0.42 | CUTA | ENSG00000112514.15 | 0.36 |
| FAM173A | ENSG00000103254.9 | 0.42 | LRRC29 | ENSG00000125122.14 | 0.36 |
| PHPT1 | ENSG00000054148.17 | 0.41 | HAGH | ENSG00000063854.12 | 0.36 |
| STUB1 | ENSG00000103266.10 | 0.41 | DDRGK1 | ENSG00000198171.12 | 0.35 |
| FDX1L | ENSG00000267673.6 | 0.41 | PFDN5 | ENSG00000123349.13 | 0.35 |
| MPND | ENSG00000008382.15 | 0.41 | DGCR6L | ENSG00000128185.9 | 0.35 |
| BAD | ENSG00000002330.13 | 0.4 | GNPTG | ENSG00000090581.9 | 0.35 |
| NDUFB10 | ENSG00000140990.14 | 0.4 | NDUFC1 | ENSG00000109390.11 | 0.35 |
| COX14 | ENSG00000178449.8 | 0.4 | SMPD1 | ENSG00000166311.9 | 0.35 |
| BLOC1S1 | ENSG00000135441.7 | 0.4 | SCRN2 | ENSG00000141295.13 | 0.35 |
| NME3 | ENSG00000103024.7 | 0.4 | WBP1 | ENSG00000239779.6 | 0.35 |
| PIGQ | ENSG00000007541.14 | 0.39 | ZNF688 | ENSG00000229809.8 | 0.35 |
| OCEL1 | ENSG00000099330.8 | 0.39 | S100A13 | ENSG00000189171.13 | 0.35 |
| SIL1 | ENSG00000120725.12 | 0.39 | NDUFB7 | ENSG00000099795.6 | 0.35 |
| ARL6IP4 | ENSG00000182196.13 | 0.39 | THAP3 | ENSG00000041988.15 | 0.35 |
| CHCHD5 | ENSG00000125611.15 | 0.39 | TCEB2 | ENSG00000103363.14 | 0.35 |
| RNF181 | ENSG00000168894.9 | 0.39 | B9D1 | ENSG00000108641.14 | 0.34 |
| SDSL | ENSG00000139410.14 | 0.39 | DPM3 | ENSG00000179085.7 | 0.34 |
| MED16 | ENSG00000175221.14 | 0.39 | EIF4EBP3 | ENSG00000243056.1 | 0.34 |
| CHMP2A | ENSG00000130724.8 | 0.39 | AES | ENSG00000104964.14 | 0.34 |
| C12orf10 | ENSG00000139637.13 | 0.39 | TMEM115 | ENSG00000126062.3 | 0.34 |
| GPX1 | ENSG00000233276.3 | 0.39 | TCTN1 | ENSG00000204852.15 | 0.34 |
| NDUFAF3 | ENSG00000178057.14 | 0.38 | FKBP2 | ENSG00000173486.12 | 0.34 |
| NDUFB2 | ENSG00000090266.12 | 0.38 | NDUFA7 | ENSG00000267855.5 | 0.34 |
| TRAPPC5 | ENSG00000181029.8 | 0.38 | TRPT1 | ENSG00000149743.13 | 0.34 |
| C19orf25 | ENSG00000119559.15 | 0.38 | GABARAP | ENSG00000170296.9 | 0.34 |
| HINT2 | ENSG00000137133.10 | 0.38 | NAA38 | ENSG00000183011.13 | 0.34 |
| TEX264 | ENSG00000164081.12 | 0.38 | EDF1 | ENSG00000107223.12 | 0.34 |
| ATP5D | ENSG00000099624.7 | 0.38 | KLHDC9 | ENSG00000162755.13 | 0.34 |
| MRPL55 | ENSG00000162910.18 | 0.38 | GFER | ENSG00000127554.12 | 0.34 |
| FAM195A | ENSG00000172366.19 | 0.38 | POLR2L | ENSG00000177700.5 | 0.34 |
| CH17-340M24.3 | ENSG00000197180.2 | 0.38 | ABHD14A | ENSG00000248487.8 | 0.34 |
| HCFC1R1 | ENSG00000103145.10 | 0.38 | CTB-113I20.2 | ENSG00000271737.1 | 0.34 |
| SURF1 | ENSG00000148290.9 | 0.37 | RP1-178F10.1 | ENSG00000260647.1 | 0.33 |
| NDUFA3 | ENSG00000170906.15 | 0.37 | 2-Mar | ENSG00000099785.10 | 0.33 |
| FIS1 | ENSG00000214253.8 | 0.37 | AC006942.4 | ENSG00000269194.1 | 0.33 |
